# The expression of CXCL13 and its relation to unfavorable clinical characteristics in young breast cancer

**DOI:** 10.1186/s12967-015-0521-1

**Published:** 2015-05-20

**Authors:** Lujia Chen, Zhongxi Huang, Guangyu Yao, Xiaoming Lyu, Jinbang Li, Xiaolei Hu, Yahong Cai, Wenji Li, Xin Li, Changsheng Ye

**Affiliations:** Breast Center, Nanfang Hospital, Southern Medical University, Guangzhou, Guangdong 510515 People’s Republic of China; Cancer Research Institute and the Provincial Key Laboratory of Functional Proteomics, Southern Medical University, Guangzhou, Guangdong 510515 People’s Republic of China; Department of Laboratory Medicine, the Third Affiliated Hospital, Southern Medical University, Guangzhou, Guangdong 510630 People’s Republic of China

## Abstract

**Background:**

Young breast cancer occupies a higher and higher proportion of breast cancer, especially in Asia, and is associated with a more unfavorable prognosis compared with the disease arising in older women. However, the poor prognosis of young breast cancer cannot be fully explained by the clinical and molecular factors.

**Methods:**

This study investigated 1125 Chinese breast cancer patients diagnosed from 2009 to 2013. A data mining of gene expression profiles was performed for the young and older breast cancer patients, identifying significantly differentially expressed genes. Quantitative RT-PCR, Western blotting and immunohistochemistry assay were carried out for the clinical sample validations.

**Results:**

The investigation firstly displayed that young patients (≤45 years) accounted for 47.6 % (535/1125) of breast cancer, and clinically associated with some unfavorable factors related to poor prognosis, such as invasive pathological type, high tumor grade, lymph node positive, ER negative and triple-negative subtype. Subsequently, 553 significantly differentially expressed genes were identified by the data mining. Of them, a set of genes related to immune function were observed to be up-regulated in young patients with breast cancer. Impressively, the CXCL13 (C-X-C motif chemokine 13) expression level showed the most significant difference (FC = 2.64, P = 8.2 × 10^−4^). Furthermore, the validations with clinical samples and correlation analysis demonstrated that CXCL13 was indeed highly expressed in young breast cancer and closely associated with some prognostic factors including lymph node positive and ER negative.

**Conclusion:**

This is the first to indicate the clinical relevance of CXCL13 to young breast cancer and represents a potential therapeutic target for young breast cancer.

**Electronic supplementary material:**

The online version of this article (doi:10.1186/s12967-015-0521-1) contains supplementary material, which is available to authorized users.

## Introduction

Breast cancer is currently one of the most common malignancies and a leading cause of cancer death in women worldwide, accounting for 23 % (1.38 million) of all new cancer cases and 14 % (458,400) of all cancer deaths [1]. It is among American women in 2014 that breast cancer is expected to account for 29 % (232,670) of new cancers, being the highest incidence of women cancer, and 15 % (40,000) of the total cancer deaths, being second only to lung cancer [2]. Although the incidence and mortality of breast cancer in Asia is lower than that of Western countries, the incidence of young breast cancer is much higher [3–6]; young breast cancer accounts for 10 % to 25 % [6, 7] of all female breast cancer in Asia, even 45 % in Saudi Arabia [8]. It is worth noting that breast cancer is a malignant tumor that mainly affects post-menopausal women, but the patients with breast cancer are getting younger and younger in recent years [4, 9] and young breast cancer accounts for 14 % of all young malignant tumor [10], and 7 % of all breast cancer [11].

Besides occupying a higher proportion of breast cancer, another reason why we should turn our attention to young breast cancer is its more aggressive biological behavior and clinical association with a more unfavorable prognosis compared with the disease arising in older women [12]. Young women with breast cancer tend to have more advanced tumor TNM staging, more invasive pathological type, higher tumor grade, higher lymph node positivity, higher proportion of triple-negativity, higher HER2 expression and lower ER/PR positivity [13–22]. A multivariate analysis [23], including the age at diagnosis, tumor size, lymph node status, tumor grade, year of treatment, protocol allocation, and expected mortality, found that even if multiple impact factors were adjusted, the breast cancer diagnosed at a young age was closely associated with an increased risk of death. Weber-Mangal *et al.* has observed some specific chromosome aberrations (such as loss of 8p22-p23 and gain of 8q23-q24) in patients with early onset breast cancer using comparative genomic hybridization (CGH), revealing that alterations in these genomic regions might be responsible for the reduced survival of patients [24]. However, the poor prognosis of young breast cancer cannot be fully explained by these clinical and molecular factors and young age is still an independent predictor of prognosis for this disease [25].

In the present study, we performed a retrospective analysis that compared a series of clinicopathological features between young and older women with breast cancer and utilized the data from GEO database to investigate the gene expression pattern of young breast cancer in Asia, expecting to know more about the potential mechanisms and help to improve the prognosis of this disease.

## Materials and methods

### Patients and clinical specimens

The complete clinical data of 1125 patients diagnosed with breast cancer were collected. These patients accepted surgical operation and treatment at Nanfang Hospital, Southern Medical University in China between October 2009 and November 2013. According to Anders *et al.’s* definition that patients aged ≤45 years were regarded as young breast cancer [26], 535 individuals were included in the young women group of our study. Patients aged ≥65 years (n = 74) were allocated to the comparison group (older women group), which represented the elder, post-menopausal women. The remaining 516 patients between 45 and 65 years of age were not involved in our analysis because our objective was to compare breast cancer arising at the extremes of age.

Additionally, a consecutive series of breast cancer specimens were collected from primary tumor of 152 patients (n = 130, ≤45 years; n = 22, ≥65 years) who did not accept neo-adjuvant chemotherapy but underwent breast-conserving surgery or modified radical mastectomy between January 2012 and August 2013. Breast cancer tissue and its corresponding adjacent normal breast tissue were obtained from each patient after excision by a surgeon and was immediately stored in liquid nitrogen until subsequent isolation of RNA and protein. This study had received approval from the Ethics Committee and all patients signed informed consents.

### Dataset and microarray analysis

Two publicly-available datasets, GSE45255 [27] and GSE15852 [28], were downloaded from the Gene Expression Omnibus (National Center for Biotechnology Information, Bethesda, MD, USA). The breast cancer samples in GSE45255 were derived from the Institute Jules Bordet (IJB; Belgium), John Radcliffe Hospital (JRH; Oxford) and the National University Hospital (NUH, Singapore). The Singapore samples, comprising 74.2 % Chinese, 13.4 % Malays and 9.2 % Indians, were utilized for further analysis. The breast caner samples in GSE15852 were collected from Malaysia, comprising 67.4 % Malays, 24.6 % Chinese and 7.3 % Indians. After screening, 36 young patient samples and 21 older patient samples assayed by Affymetrix U133A GeneChips were used in the present study. The details of the datasets are presented in Additional file 1: Table S1.

Using R software package, gene expression profiling data was re-summarized by the RMA method [29] and Entrez gene-centric CDF files [30] (instead of original Affymetrix CDF files), which filtered out non-specific probes on the GeneChips and merged multiple probe sets representing the same Entrez gene into one probe set. The combat algorithm (CA) [31] was adopted to eliminate the batch effect of microarray data because these data were from two different batches of experiments. Significance analysis of microarray (SAM) [32] was performed to identify differentially expressed genes between young and older breast cancer tissues. Delta was set to 0.6, and the threshold of FDR was set to 0.182. The differentially expressed genes were further analyzed with GenCLiP on-line software [33, 34] (http://ci.smu.edu.cn) to annotate gene functions and perform KEGG Pathway analysis.

### Extraction of total RNA and quantitative RT-PCR

Total RNA was extracted from tissue samples with TRIzol (Invitrogen) according to the user’s manual. Reverse transcription was performed using PrimeScript™ RT reagent Kit with gDNA Eraser (Perfect Real Time) (TaKaRa Code NO. RR047) and was run at 42 °C for 2 min to remove genomic DNA, then 37 °C for 15 min and 85 °C for 5 s. Real-time PCR was performed using SYBR® Premix Ex Taq™(Tli RNaseH Plus) (TaKaRa Code NO. RR420A) on an Mx3005P (Stratagene) with 10 min at 95 °C, 45 cycles of 10 s at 95 °C, 10 s at 60 °C, and 15 s at 72 °C (data capture), and finally melting profile analysis (55 °C −95 °C). Primer sequences were either derived from Primer Bank [35] or designed using primer5 primer design software (ESR1 forward primer: 5′-GGGAAGTATGGCTATGGAATCTG-3′, ESR1 reverse primer: 5′-TGGCTGGACACATATAGTCGTT-3′. GABRP forward primer: 5′-TTTCTCAGGCCCAATTTTGGT-3′, GABRP reverse primer: 5′- GCTGTCGGAGGTATATGGTGG-3′. CXCL13 forward primer: 5′-GCTTGAGGTGTAGATGTGTCC-3′, CXCL13 reverse primer: 5′-CCCACGGGGCAAGATTTGAA-3′. GAPDH forward primer: 5′- CTGCACCACCAACTGCTT-3′, GAPDH reverse primer: 5′- TTCTGGGTGGCAGTGATG-3′). GAPDH was employed to normalize the expression of target gene. The relative quantification (Fold Change) between different samples was compared between cancer and normal sample as well as between young women group and older women group according to the 2^−∆∆Ct^ method as described by Livak and Schmittgen [36]. Quantitative RT-PCR (qRT-PCR) was conducted for each sample in triplicate.

### Western blotting

Protein was extracted from cancer tissue and adjacent normal breast tissue using RIPA buffer with protease inhibitors and quantified using the BCA protein assay kit (Thermo Scientific, America). Protein (20 μg) was loaded onto a 12 % SDS–PAGE gel that was then transferred onto a PVDF membrane and incubated with rabbit monoclonal CXCL13 antibody (1:500; Abcam) at 4 °C overnight in blocker (3 % non-fat dry milk/BSA in TTBS). After washing, the membrane was incubated with mouse anti-rabbit HRP-conjugated secondary antibody (1:3,000; ProteinTech) for 2 h at room temperature. Protein was normalized with GAPDH (1:3,000; ProteinTech) and measured by densitometry using ECL detection (Bio-Rad, America).

### Immunohistochemistry assay

Immunohistochemical staining was performed on formalin-fixed, paraffin-embedded tissue sections, which had been obtained for a routine diagnostics using standard techniques. The slides were dewaxed into xylene and rehydrated through graded alcohols. Following immersion in citrate buffer for antigen retrieval under pressure, the slides were placed in 3 % H_2_O_2_ for 15 min. The primary CXCL13 or CD45 antibody (Abcam) (diluted 1:20 in 3 % BSA/PBS) was incubated on the slides at 4 °C overnight. After washed in PBS, the mouse anti-rabbit HRP-conjugated secondary antibody (ProteinTech) was applied for 30 min. The slides were next incubated with DAB for 5 min and counterstained with 20 % hematoxylin, dehydrated, cleared and mounted.

### Statistical analysis

All statistical analyses were performed by the SPSS 20.0 statistical software package (SPSS Inc. Chicago, IL, USA). *χ*^2^ test and Mann–Whitney *U* test were employed to determine the differences of clinicopathological characteristics between young and older women groups. Wilcoxon sign test and Mann–Whitney *U* test were used to compare gene expression levels of CXCL13, GABRP and ESR1 and protein expression levels of CXCL13 between different groups. Mann–Whitney *U* test was used for the comparison of the immunohistochemistry results of CXCL13. Kruskal-Wallis H test, Mann–Whitney *U* test and linear regression analysis were used to analyze the relationship between CXCL13 expression level and clinicopathological characteristics. Mann–Whitney *U* test was used to compare the differences of clinicopathological characteristics between the groups with relatively low and high CXCL13 expression. The results were considered significant if P < 0.05.

## Results

### Clinicopathological characteristics of young breast cancer

A total of 1125 women with age ranging from 20 to 87 years and a median of 46 years were diagnosed with breast cancer at Nanfang Hospital, Southern Medical University in China. Of them, 535 patients (47.6 %) were younger than or equal to 45 years and 74 patients (6.6 %) were older than or equal to 65 years.

The clinicopathological characteristics were comparatively analyzed between young women group (≤45 years) and older women group (≥65 years). As shown in Table 1, significant differences occurred in pathological type (P = 0.001), tumor grade (P = 0.009), lymph node status (P = 0.035), ER status (P = 0.041) and molecular subtypes (P = 0.005) between the two groups. The proportion of IDC-NOS (invasive ductal carcinoma, not otherwise specified) was higher in young women group than in older women group (85.4 % versus 72.9 %), whereas the proportion of medullary carcinoma, mucinous carcinoma and other special types of invasive carcinoma was lower in young women group than in older women group (5.1 % versus 18.9 %). The proportion of luminal A subtype in young women group was lower than that of older women group (9.9 % versus 24.3 %), while the proportion of triple-negative subtype was higher in young women group (14.9 % versus 8.1 %). Moreover, compared with their older counterparts, tumor grade, lymph node positive (45.8 % versus 32.4 %) and ER negative (37.7 % versus 25.7 %) were relatively higher in young group, but no difference was observed in tumor size, PR status, HER2 status and TNM staging.

**Table 1:** Clinical characteristic by age

Characteristic	Patients with breast cancer				P
	Young (≤45 ys)		Old (≥65 ys)		
	(n = 535)		(n = 74)		
	No.	%	No.	%	
Age, years					---
Range	20–45		65–87		
Median	40		69		
Pathology					0.001
IDC-NOS	457	85.4	54	72.9	
ILC	16	3.0	3	4.1	
DCIS	35	6.5	3	4.1	
Others*	27	5.1	14	18.9	
Tumor size, mm					0.463
Range	5–150		8–100		
Median	24		25		
Tumor grade					0.009
1	44	8.2	14	18.9	
2	302	56.5	38	51.3	
3	121	22.6	11	14.9	
Missing	68	12.7	11	14.9	
Lymph node status					0.035
Positive	245	45.8	24	32.4	
Negative	288	53.8	49	66.2	
Missing	2	0.4	1	1.4	
ER status					0.041
Positive	331	61.9	55	74.3	
Negative	202	37.7	19	25.7	
Missing	2	0.4	---	---	
PR status					0.739
Positive	349	65.2	47	63.5	
Negative	184	34.4	27	36.5	
Missing	2	0.4	---	---	
HER2 status					0.068
Negative, 0-1+	291	54.4	49	66.2	
Equivocal, 2+	122	22.8	13	17.6	
Positive, 3+	119	22.2	12	16.2	
Missing	3	0.6	---	---	
TNM staging					0.762
0	34	6.4	4	5.4	
I	134	25.1	21	28.4	
II	219	40.9	29	39.2	
III	120	22.4	16	21.6	
IV	8	1.5	1	1.3	
Missing	20	3.7	3	4.1	
Subtypes					0.005
Luminal A	53	9.9	18	24.3	
Luminal B	269	50.3	36	48.7	
HER2	69	12.9	10	13.5	
Triple negative	80	14.9	6	8.1	
Missing	64	12.0	4	5.4	

*Abbreviations*: *IDC-NOS*, invasive ductal carcinoma, not otherwise specified; *ILC*, invasive lobular carcinoma; *DCIS*, ductal carcinoma in situ

*Medullary carcinoma, mucinous carcinoma, tubular carcinoma, invasive papillary carcinoma and other special types of invasive carcinoma

### Differential gene expression pattern of young breast cancer

**Fig. 1:**
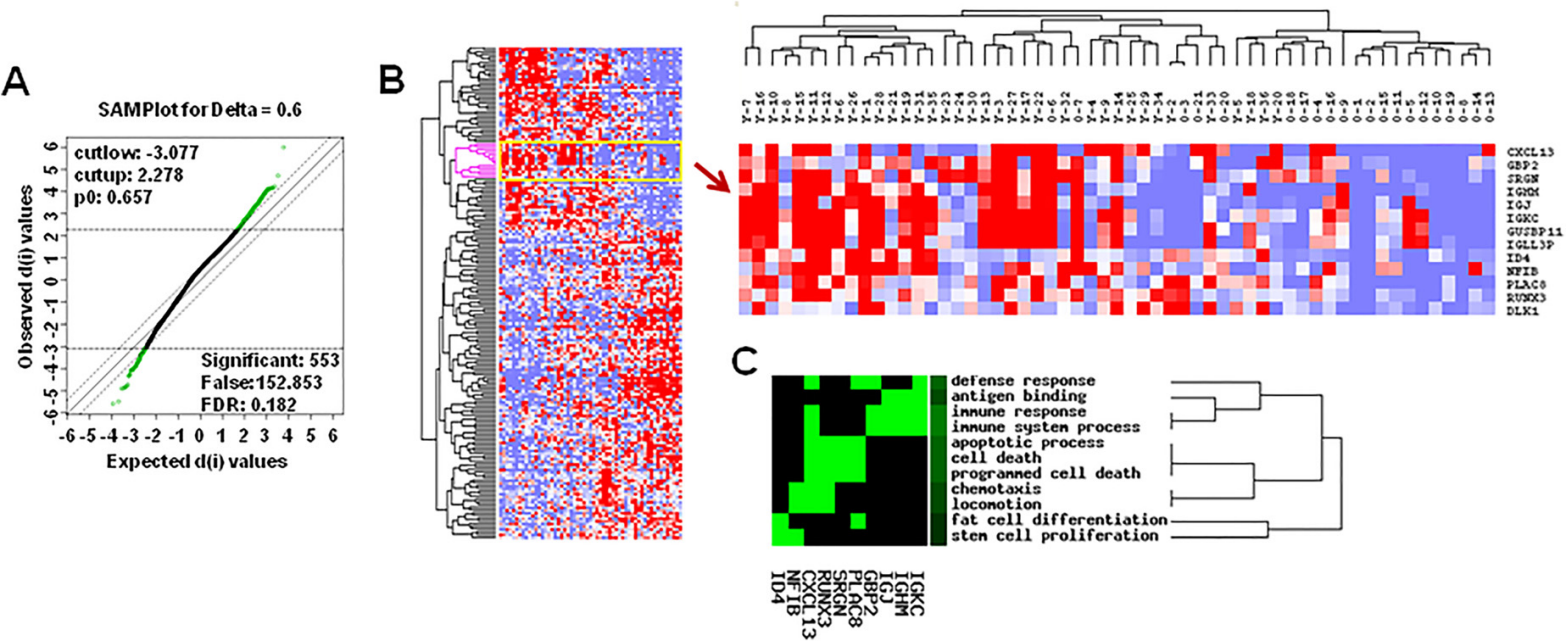
Data mining of Gene expression profiles. **a** Significance analysis of microarray (SAM) was performed to identify differentially expressed genes between young and older breast cancer tissues. Delta was set to 0.6, and the threshold of FDR was set to 0.182. **b** Supervised hierarchical clustering of 553 differentially expressed genes. The heat map revealed the gene expression patterns between young patients and older patients. All samples were denoted in columns and genes were denoted in rows (gene symbols for a cluster of genes were listed on the right and the details of all differentially expressed genes could be found in Additional file 2: Table S2). The mapped expression levels for all genes were depicted using a color scale; highly expressed genes were indicated in red and lowly expressed in blue. **c** GO analysis of a cluster of differentially expressed genes was performed with GenCLiP software (http://ci.smu.edu.cn)

Based on two microarray datasets (GSE45255 and GSE15852) downloaded from GEO, 57 Asian breast cancer tissues collected from 36 young and 21 older patients were re-analyzed. SAM analysis showed 553 significantly differentially expressed genes between young and older breast cancer; 81 genes were up-regulated and 472 genes were down-regulated in young breast cancer (Fig. 1a & b, Additional file 2: Table S2) relative to older patients. Top 36 up-regulated genes and top 27 down-regulated genes in young breast cancer were listed in Table 2 and Table 3 respectively. Of them, a set of genes related to immune function, such as CXCL13, IGHM, IGLL3P, IGJ and IGKC, were up-regulated in young patients with breast cancer. CXCL13 expression displayed the most significant difference (FC = 2.64, P = 8.2 × 10^−4^) (Table 2). In addition, two identified genes, GABRP, positively associated with young breast cancer [37], and ESR1, down-regulated in young breast cancer [38], were also included (Tables 2 & 3).

**Table 2:** Top 36 up-regulated genes in young patients with breast cancer

NO.	Gene_symbol	Gene_name	FC	P
1	KRT14	Keratin 14	4.31	5.8 × 10^−7^
2	GABRP	Gamma-aminobutyric acid (GABA) A receptor, pi	2.82	7.2 × 10^−6^
3	PROM1	Prominin 1	2.76	2.6 × 10^−4^
4	CXCL13	Chemokine (C-X-C motif) ligand 13	2.64	8.2 × 10^−4^
5	IGHM	Immunoglobulin heavy constant mu	2.63	1.7 × 10^−4^
6	IGLL3P	Immunoglobulin lambda-like polypeptide 3, pseudogene	2.61	1.5 × 10^−3^
7	MMP7	Matrix metallopeptidase 7 (matriysin, uterine)	2.52	2.3 × 10^−4^
8	IGJ	Immunoglobulin J polypeptide	2.40	8.0 × 10^−4^
9	KRT17	Keratin 17	2.37	7.4 × 10^−7^
10	GUSBP11	Glucuronidase, beta pseudogene 11	2.35	2.0 × 10^−3^
11	KRT15	Keratin 15	2.16	1.4 × 10^−3^
12	KRT7	Keratin 7	2.14	3.1 × 10^−3^
13	TSPYL5	TSPY-like 5	2.03	5.0 × 10^−5^
14	IGKC	Immunoglobulin kappa constant	1.97	2.4 × 10^−3^
15	S100A2	S100 calcium binding protein A2	1.91	2.1 × 10^−4^
16	LDHB	Lactate dehydrogenase B	1.85	6.4 × 10^−4^
17	SFRP1	Secreted frizzled-related protein 1	1.83	5.5 × 10^−4^
18	KRT5	Keratin 5	1.79	6.1 × 10^−4^
19	CDH3	Cadherin 3, type 1, P-cadherin (placental)	1.78	9.9 × 10^−6^
20	RND3	Rho family GTPase 3	1.78	9.7 × 10^−6^
21	SYNM	Synemin, intermediate filament protein	1.76	1.8 × 10^−4^
22	KIT	V-kit Hardy-Zuckerman 4 feline sarcoma viral oncogene homolog	1.75	7.0 × 10^−6^
23	KRT6B	Keratin 6B	1.75	1.3 × 10^−4^
24	MT1X	Metallothionein 1X	1.74	1.8 × 10^−3^
25	AMIGO2	Adhesion molecule with Ig-like domain 2	1.65	2.1 × 10^−3^
26	SLC6A14	Solute carrier family 6 (amino acid transporter), member 14	1.63	4.3 × 10^−4^
27	RGS2	Regulator of G-protein signaling 2, 24 kDa	1.61	9.3 × 10^−4^
28	SRGN	Serglycin	1.60	2.9 × 10^−3^
29	DDIT4	DNA-damage-inducible transcript 4	1.60	1.4 × 10^−3^
30	NFIB	Nuclear factor I/B	1.58	1.8 × 10^−3^
31	DTX4	Deltex homolog 4 (Drosophila)	1.58	1.4 × 10^−4^
32	CSDA	Cold shock domain protein A	1.57	3.8 × 10^−4^
33	GBP2	Guanylate binding protein 2, interferon-inducible	1.54	1.8 × 10^−3^
34	ITM2A	Integral membrane protein 2A	1.53	1.9 × 10^−3^
35	PADI2	Peptidyl arginine deiminase, type II	1.52	1.4 × 10^−4^
36	RBMS1	RNA binding motif, single stranded interacting protein 1	1.52	3.2 × 10^−4^

*Abbreviations*: *FC*, Fold change

**Table 3:** Top 27 down-regulated genes in young patients with breast cancer

NO.	Gene_symbol	Gene_name	FC	P
1	NAT1	N-acetyltransferase 1 (arylamine N-acetyltransferase)	0.37	2.3 × 10^−3^
2	GRIA2	Glutamate receptor, ionotropic, AMPA 2	0.45	1.5 × 10^−2^
3	C6orf211	Chromosome 6 open reading frame 211	0.49	9.9 × 10^−3^
4	PSD3	Pleckstrin and Sec7 domain containing 3	0.49	6.1 × 10^−4^
5	GFRA1	GDNF family receptor alpha 1	0.49	3.4 × 10^−3^
6	EVL	Enah/Vasp-like	0.50	1.0 × 10^−3^
7	SCUBE2	Signal peptide, CUB domain, EGF-like 2	0.51	2.2 × 10^−2^
8	DNAJC12	DnaJ (Hsp40) homolog, subfamily C, member 12	0.52	1.9 × 10^−3^
9	KCNE4	Potassium voltage-gated channel, Iskrelated family, member 4	0.52	7.5 × 10^−3^
10	ECM1	Extracellular matrix protein 1	0.52	1.8 × 10^−3^
11	TBC1D9	TBC1 domain family, member 9 (with GRAM domain)	0.53	4.6 × 10^−4^
12	CYP2B7P1	Cytochrome P450, family 2, subfamily B, polypeptide 7 pseudogene 1	0.53	7.3 × 10^−3^
13	ESR1	Estrogen receptor 1	0.54	8.3 × 10^−8^
14	CA12	Carbonic anhydrase XII	0.55	1.2 × 10^−3^
15	MYB	V-myb myeloblastosis viral oncogene homolog (avian)	0.56	1.4 × 10^−2^
16	PLAT	Plasminogen activator, tissue	0.59	2.7 × 10^−2^
17	PGR	Progesterone receptor	0.59	1.6 × 10^−2^
18	CLGN	Calmegin	0.60	2.5 × 10^−3^
19	SLC44A4	Solute carrier family 44, member 4	0.61	8.1 × 10^−3^
20	REEP1	Receptor accessory protein 1	0.62	9.6 × 10^−4^
21	AR	Androgen receptor	0.64	9.7 × 10^−4^
22	F7	Coagulation factor VII (serum prothrombin conversion accelerator)	0.64	2.1 × 10^−3^
23	CCDC170	Coiled-coil domain containing 170	0.64	1.3 × 10^−4^
24	SELENBP1	Selenium binding protein 1	0.65	2.3 × 10^−2^
25	PRKAR2B	Protein kinase, cAMP-dependent, regulatory, type II, beta	0.65	2.0 × 10^−3^
26	GLCE	Glucuronic acid epimerase	0.66	2.0 × 10^−4^
27	RNASE4	Ribonuclease, RNase A family, 4	0.66	9.5 × 10^−4^

*Abbreviations*: *FC*, Fold change

Furthermore, 553 differentially expressed genes were analyzed by GenCLiP software. Notably, GO analysis revealed that CXCL13 was clustered into a small group of genes that were involved in a variety of cellular functions such as immune response, immune system process, cell death, programmed cell death and so on (Fig. 1b & c). KEGG Pathway analysis showed the involvement of several signal pathways, such as calcium-, insulin-, Wnt-signalling and so on (Additional file 3: Figure S1).

Therefore, our data suggest that CXCL13 may be an unidentified gene associated with young breast cancer and deserves further investigation.

### High CXCL13 mRNA and protein expression in young breast cancer

**Fig. 2:**
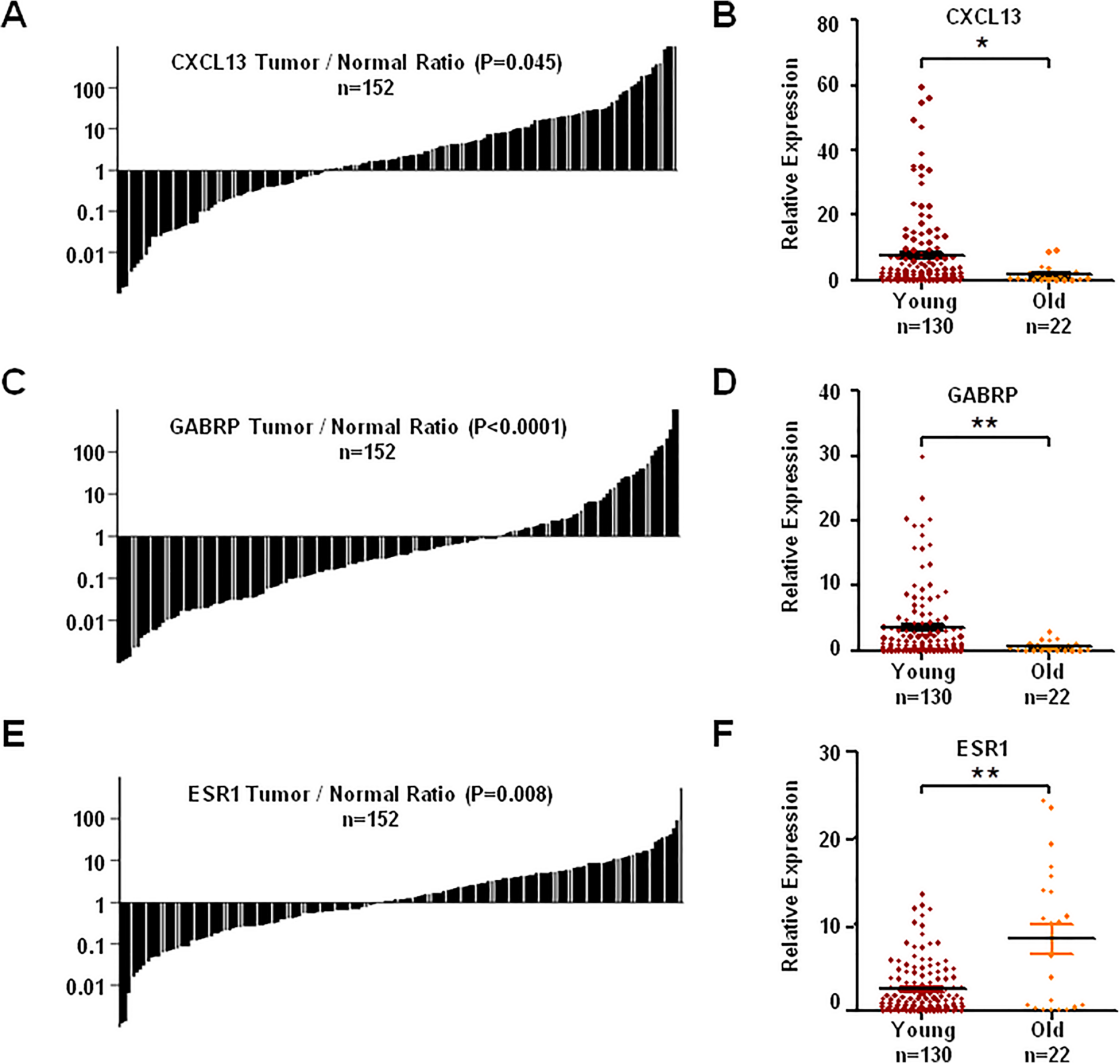
Real-time PCR validation of mRNA expression in patients with breast cancer. **a**, **c**, **e** The ratios of CXCL13, GABRP and ESR1 mRNA expression in cancer tissues to their corresponding adjacent normal tissues were calculated after real-time PCR detection and normalization to GAPDH expression. X-axis indicates the ratio of mRNA expression in the cancer tissues to their corresponding adjacent normal tissues and Y-axis indicates the number of the specimens. **b**, **d**, **f** Expression levels of CXCL13, GABRP and ESR1 in young women group (n = 130) and older women group (n = 22) were analyzed by real-time PCR analysis and normalized to GAPDH expression. *P < 0.05, **P < 0.01

To further validate our results, quantitative RT-PCR was performed to detect the expression levels of related genes in 152 pairs of breast cancer tissues and their corresponding adjacent normal tissues (n = 130, young women group; n = 22, older women group). The results displayed that CXCL13 mRNA expression was significantly up-regulated in 63.2 % of breast cancer tissues (96 in 152, P = 0.045) and indeed increased in young patients’ tissues compared with older counterparts (P = 0.011). Interestingly, qPCR also showed that GABRP expression was down-regulated (67.8 %, 103 in 152, P < 0.0001) and ESR1 expression was up-regulated (53.3 %, 81 in 152, P = 0.008) in these cancer tissues compared with their corresponding adjacent normal tissues. Particularly, GABRP increased (P = 0.005) while ESR1 decreased (P = 0.009) in young breast cancer (Fig. 2).

**Fig. 3:**
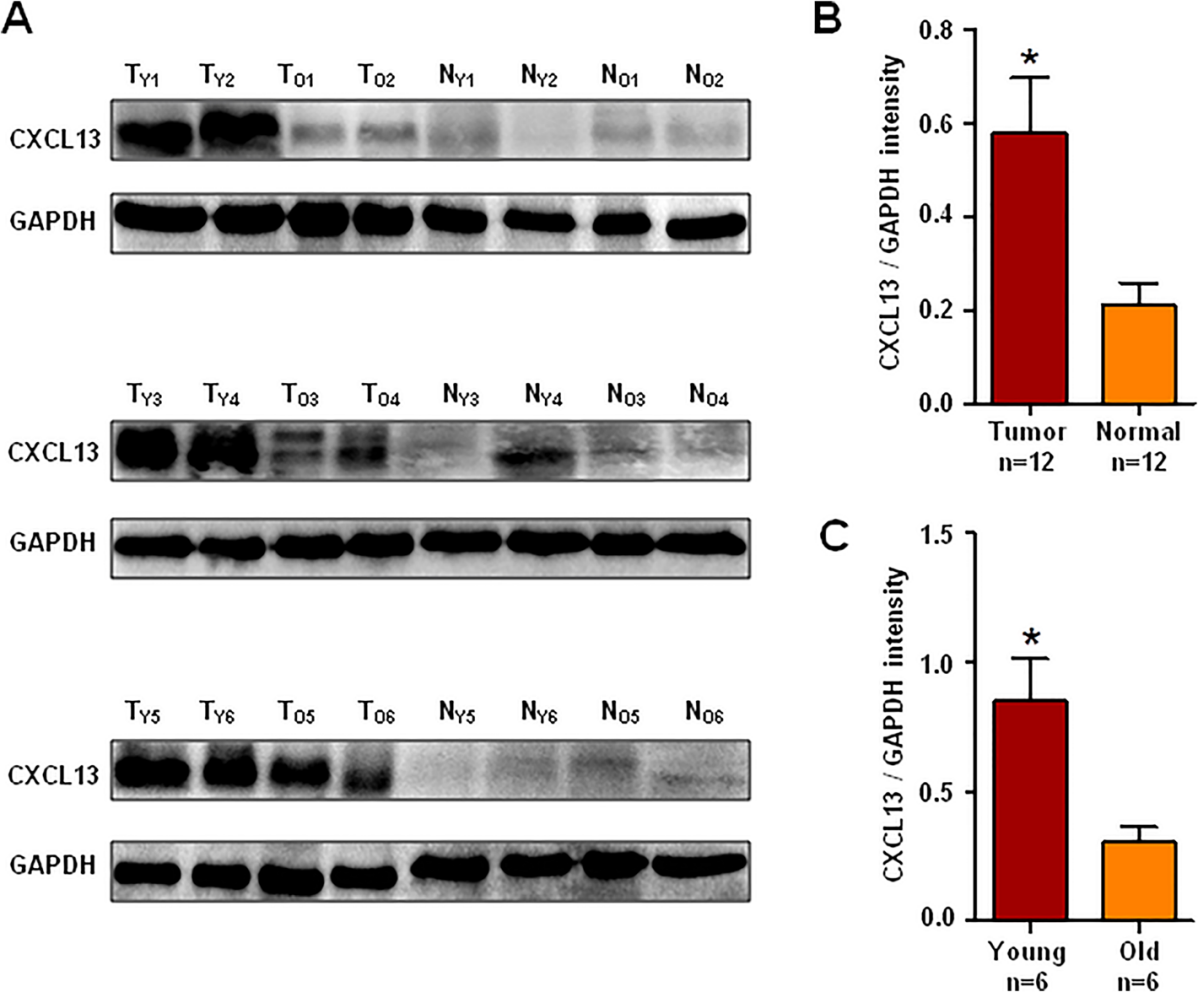
Western blotting detection of CXCL13 protein in patients with breast cancer. **a** Expression levels of CXCL13 protein were assessed by Western blotting analysis and normalized to GAPDH. T_Y1–6_ and T_O1–6_ represented randomly selected cancer tissues from young women group and older women group, respectively, and N_Y1–6_ and N_O1–6_ represented the randomly selected corresponding adjacent normal tissues from young women group and older women group, respectively. **b** A semi-quantitative analysis of the Western blotting of CXCL13 protein was performed between cancer tissues (n = 12) and their corresponding adjacent normal tissues (n = 12). **c** A semi-quantitative analysis of the Western blotting of CXCL13 protein in cancer tissues between young patients (n = 6) and their older counterparts (n = 6). *P < 0.05

Additionally, 12 pairs of specimen tissues (n = 6, young women group; n = 6, older women group) were randomly selected for the detection of CXCL13 protein expression by Western blotting. The results displayed that CXCL13 protein expression was significantly higher in cancer tissues than that of their corresponding adjacent normal tissues (P = 0.015) and significantly up-regulated in young patients’ tissues relative to older counterparts (P = 0.041) (Fig. 3).

**Fig. 4:**
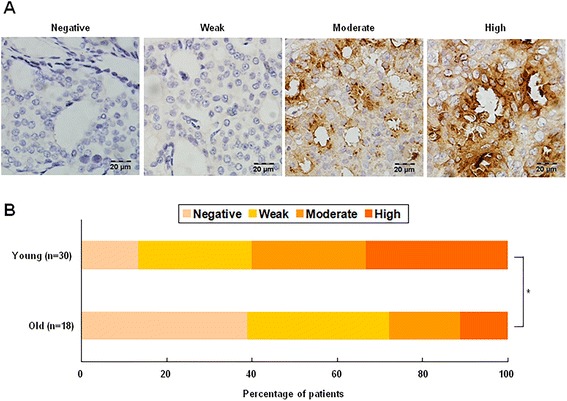
Immunohistochemistry detection of CXCL13 protein in patients with breast cancer. **a** Representative IHC of breast cancer samples, showing the negative, weak, moderate and high expression level of CXCL13, respectively. **b** The percentage of patients with the negative, weak, moderate and high expression of CXCL13 protein in young and older women group. *P < 0.05

IHC staining of CXCL13 was next conducted in 48 paraffin sections randomly selected from formalin-fixed and routinely processed breast cancer tissues (n = 30, young women group; n = 18, older women group). The protein expression levels were scored as 0, 1, 2 and 3 for the negative, weak, moderate and high expression of CXCL13, respectively. We did observe that CXCL13 protein was expressed in young breast cancer in a higher level than that of their older counterparts (P = 0.015) (Fig. 4b, Table 4). It was expressed in tumor cells but not stromal cells (CD45 positive cells) (Fig. 4a, Additional file 4: Figure S2 & Additional file 5: Figure S3).

**Table 4:** Expression status of CXCL13 in patients with breast cancer by immunohistochemistry

CXCL13	Patients with breast cancer		P
	Young (≤45 years) (n = 30)	Old (≥65 years) (n = 18)	
0	4	7	0.015
1	8	6	
2	8	3	
3	10	2	

Collectively, these data indicated a high CXCL13 expression level in young breast cancer.

### The correlation of high CXCL13 expression with clinicopathological features

**Fig. 5:**
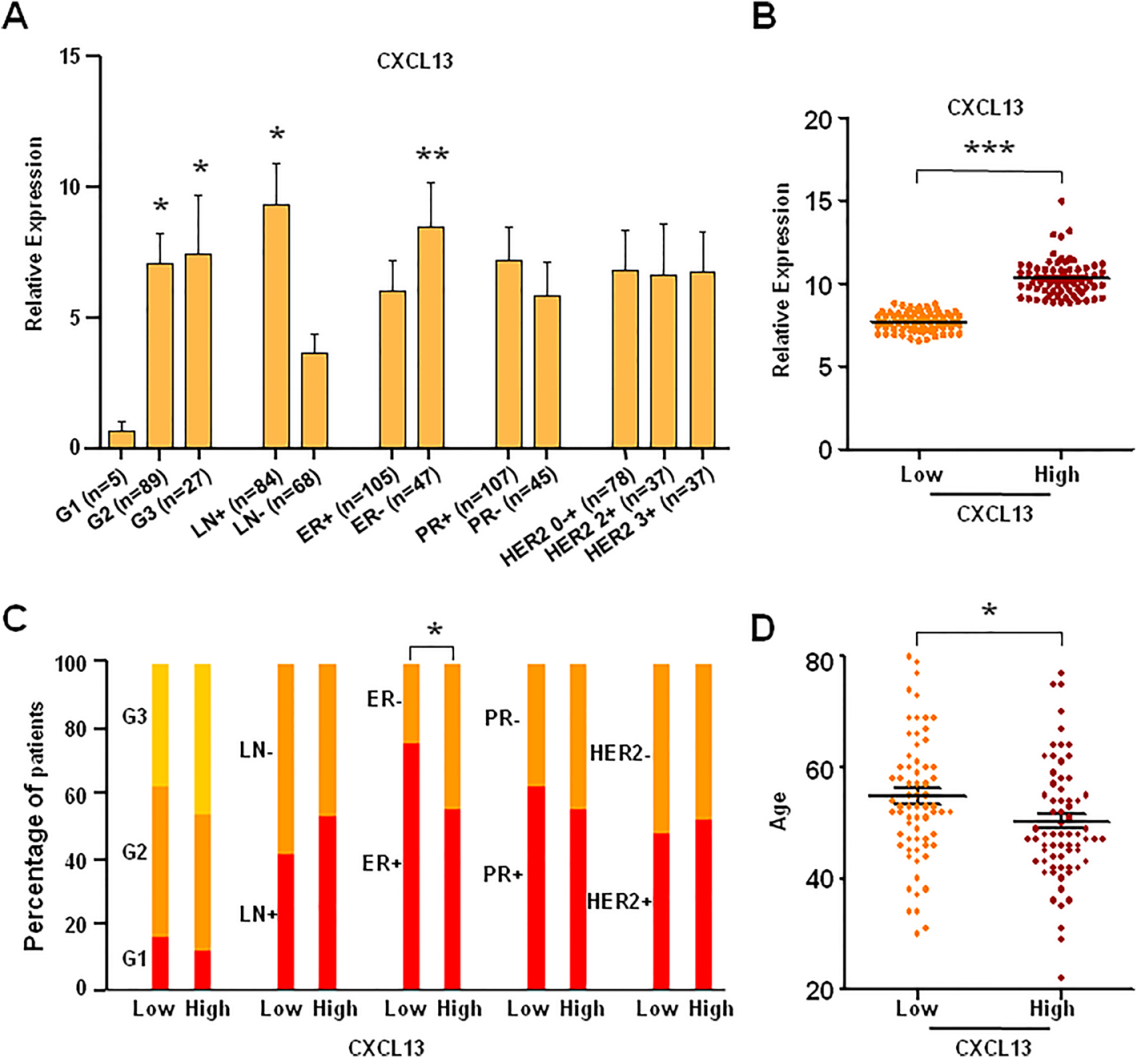
Analysis of the correlation of CXCL13 expression with clinicopathological features. **a** The CXCL13 mRNA expression in 152 clinical tissue specimens was compared according to clinicopathological features. **b**, **c**, **d** 138 microarrays from GSE45255 and GSE15852 were divided into two groups according to their relative expression levels of CXCL13 (low expression group and high expression group). The CXCL13 expression and clinicopathological features were compared between these two groups. *P < 0.05, **P < 0.01, ***P < 0.001

To explore the potential significance of CXCL13 in young breast cancer, correlation analysis was performed in 152 clinical tissue specimens with breast cancer to assess the association of CXCL13 expression with clinicopathological features, including tumor grade, lymph node status, ER status, PR status and HER2 status. Notably, the results showed that CXCL13 mRNA expression was obviously correlated with tumor grade, lymph node status or ER status; CXCL13 mRNA expression was significantly up-regulated in grade 2/3 (P = 0.046/ P = 0.035), lymph node positive (P = 0.012) or ER negative group (P = 0.005) (Fig. 5a). Furthermore, linear regression analysis showed that age, lymph node status and ER status, were the independent factors of CXCL13 mRNA expression (Additional file 6: Table S3).

We also divided 138 microarrays from GSE45255 and GSE15852 into two groups according to their relative expression levels of CXCL13 (low expression group and high expression group, as shown in Fig. 5b). Upon comparing with low expression group, the significantly higher proportion of ER negative and younger patients appeared in the high expression group (Fig. 5c & d). In addition, grade 3 and lymph node positive could be more frequently observed in the high CXCL13 expression group (Fig. 5c, Additional file 7: Figure S4) though no statistically significant differences were found.

## Discussion

Breast cancer is a serious disease that affects the physical and mental health of women. In recent years, patients with breast cancer tend to be younger and the incidence of young breast cancer is increasing gradually [4, 9, 39]. In particular, the incidence and proportion of young breast cancer are much higher in Asia than in Western countries [3–8], suggesting the differences in the incidence of young breast cancer between different ethnic groups. In the present study, 1125 breast cancer patients were collected from October 2009 and November 2013 and 535 patients were observed to be younger than or equal to 45 years, occupying 47.6 % of all the patients. This is consistent with the data from Saudi Arabia [8], providing additional evidence for the higher proportion of young breast cancer in Asia.

A retrospective analysis was performed in a large cohort of patients to compare the differences between young women group (≤45 years) and older women group (≥65 years). Firstly, we found a statistical difference in pathological types between these two groups; invasive cancer types were more frequently observed in young patient group. Secondly, we confirmed the higher tumor grade, higher lymph node positivity, and lower ER positivity in young breast cancer. These data indicated that young breast cancer was more aggressive and probably associated with poor prognosis. Thirdly, we observed that the pattern of molecular subtypes in young women group was obviously different from that of older women group; luminal A subtype was less and triple-negative subtype was more in young patients. However, we noticed a much lower percentage of Luminal A and a higher percentage of Luminal B patients in our cohort than that of previous reports [40]. This might be caused by our assessment of molecular subtypes based on the standard of Ki67 < 14 % or ≥14 % in the St Gallen International Expert Consensus [41] in the absence of a specific standard for Ki67 in the Department of pathology of our hospital. Fortunately, the result could display the difference in molecular subtypes between two groups, consistent with previous report [42]. A previous study reported that luminal A subtype breast cancer had the best prognosis, while the triple-negative subtype patients had the worst prognosis [40]. Thus, the unfavorable prognosis of young breast cancer may be attributed to the different constitution of molecular subtypes. However, the retrospective analysis did not show any differences in tumor size, PR status, HER2 status and TNM staging between two groups. By carefully analyzing the patient-based data, we observed that most of patients in older women group came from the villages where lacked self-care awareness and routine mammography screen, so these patients failed to see a doctor in time when they had a suspicious breast mass, easily leading to a larger tumor with more lymph node metastasis. This may be one of reasons why we could not observe a significant difference in tumor size and TNM staging between young and older women patients. Moreover, probably due to relatively fewer samples in older women group, the difference in HER2 expression between two groups did not reach statistical significance though a trend towards a higher level of HER2 expression appeared in young breast cancer.

To provide an insight into the potential mechanism of tumor biology in young breast cancer, we re-analyzed two sets of microarray data downloaded from GEO. We found 81 up-regulated genes and 472 down-regulated genes in young breast cancer compared with older breast cancer. Notably, GABRP [37] and ESR1 [38] previously reported to be related to young breast cancer were included in these differentially expressed genes and validated by qRT-PCR, proving the reliability of our data mining and allocation of samples. More interestingly, in addition to these two genes, a set of genes related to immune function, such as CXCL13, IGHM, IGLL3P, IGJ and IGKC, were observed to be significantly up-regulated in young breast cancer and CXCL13 showed the most significant difference, implying that abnormality of immune functions may be a potential risk factor for young breast cancer. Moreover, we observed that CXCL13 was clustered with some differentially expressed genes that were involved in a variety of cellular functions such as immune response, immune system process, cell death, programmed cell death and so on by GO analysis, implying that CXCL13 may play multiple roles in young breast cancer.

The functions of CXC-chemokines are initially thought to be chemoattraction and activation of leukocytes in diverse immunological responses [43], but nowadays the important roles of CXC-chemokine ligands and their corresponding receptors in neoplastic transformation, cancer cell migration, invasion, and metastasis have been proved by increasing evidences [44–47]. For example, CXCL12 promoted cell migration, cell growth, and invasion of ovarian cancer cells [48]. CXCR1 and CXCR2 could stimulate prostate cancer progression through autocrine signaling of cancer cells [46]. CXCL1 and CXCL8 could act as autocrine growth factors [49–51]. CXCL13 is one of important chemokines. It is also known as BLC or BCA1 [52, 53] and is a marker of B lymphocyte aggregation, playing a key role in homing, migration and accumulation of B lymphocyte [52] by specially binding with CXCR5 [54]. The impact of CXCL13/CXCR5 on various types of cancers including breast cancer has recently attracted much interest [55–60]. Depending on PI3Kp110, Src and FAK, the interaction of CXCR5 with its specific ligand—CXCL13, could promote prostate cancer cell invasion, migration, and differential matrix metalloproteinase (MMP) expression [59]. Panse *et al.*’s microarray analysis, followed by the validation in breast cancer samples and cell lines, revealed an overexpressed CXCL13 in breast cancer tissues. Panse *et al.* detected a significantly elevated serum concentration of CXCL13 in breast cancer patients with metastatic disease as compared with controls and disease-free patients [55]. Razmkhah’s research showed a significantly high expression level of CXCR5 transcript in lymph node positive patients with stage III compared to those with stage II tumors, and a higher mRNA expression of CXCL13 in lymph node positive samples compared to lymph node negative samples though the difference was not significant [61]. A recent study provides evidence that co-expression of CXCL13 and CXCR5 shows a significant correlation with lymph node metastasis and CXCL13 has the EMT-inducing potential [62]. In the present study, to validate the possible roles of CXCL13 in young breast cancer, we carried out multiple detections of CXCL13 expression at either mRNA or protein level in a relatively large set of clinical tissue specimens from patients with breast cancer. Our results were consistent with these previous reports, indicating a highly expressed CXCL13 in breast cancer though these data may be affected by the existence of fat tissue and adenocarcinoma cells in adjacent normal breast tissue. Impressively, we further observed that this gene was highly expressed in young breast cancer compared with their older counterparts. The clinical correlation analysis and linear regression analysis supported the potential significance of CXCL13 in young breast cancer. This may be helpful for establishing a potential association between CXCL13 and young breast cancer, explaining why young patients with breast cancer are prone to develop metastasis and why young age at diagnosis are associated with poor prognosis.

However, to date there have been two different opinions; one is that immune cells in tumor environment play a primary role of tumor rejection and the up-regulation of some immune genes are related to the better prognosis [63–67]. Another is that they sometime show pro-tumor rather than anti-tumor properties in tumor microenvironment [68–70]. Similarly, there are opposite opinions or data about the role of CXCL13 in cancer. Razis *et al.* showed that activation of CXCL13/CXCR5 axis was associated with the determinants of poor prognosis but improved the outcome of the HER2 overexpressing subpopulation [71]. The good prognostic value of CXCL13, particularly in ER- and HER2+ breast cancer, was also confirmed by Gu-Trantien *et al.*[72]. These data suggests less obvious roles of CXCL13 in the poor prognosis of young breast cancer. To our knowledge, these conflicting data yielded may be due to the possibility that chemokines exert different functions in different environments or immune states influenced by many factors such as race, age, disease, and so on. Therefore, a long term and larger sample-size study in Asian people with breast cancer is deserved to further validate our data.

It has been reported that chemokines produced by solid epithelial tumors such as ovarian cancer [73] and breast cancer [74, 75], were associated with leukocyte infiltration, especially macrophage infiltration [76, 77]. Chemokines were found in colorectal cancer to be associated with the accumulation of tumor-associated macrophages (TAM) that favor tumor progression instead of normal immune functions [78]. These studies hint the existence of a possible relationship between high CXCL13 expression in young breast cancer and TAM accumulation in tumor microenvironment. We are planning to elucidate it in future.

## Conclusion

In summary, the present study investigated the proportion of young breast cancer in Asia and re-confirmed some unfavorable factors related to the poor prognosis in young breast cancer. Using the data mining of gene expression profiles and the clinical sample-based validations, we first showed that CXCL13 was highly expressed in young breast cancer, and closely associated with some prognostic factors including lymph node positive and ER negative in young breast cancer. CXCL13 may be a potential unfavorable factor for young breast cancer in Asia, though its prognostic value remains unclear.

## Additional files

Additional file 1: Table S1. Clinical characteristic of gene microarray with breast cancer. Additional file 2: Table S2. 553 differentially expressed genes identified by SAM Method. Additional file 3: Figure S1. KEGG pathway analysis of differentially expressed genes. Additional file 4: Figure S2. Immunohistochemistry detection of CXCL13 protein in patients with breast cancer. Additional file 5: Figure S3. Immunohistochemistry detection showing the location of CXCL13 protein. Additional file 6: Table S3. Linear regression analysis of the correlation of CXCL13 expression with clinicopathological features. Additional file 7: Figure S4. Analysis of the correlation of CXCL13 expression with tumor size.
