# Erratum to: The expression of CXCL13 and its relation to unfavorable clinical characteristics in young breast cancer

**DOI:** 10.1186/s12967-016-1069-4

**Published:** 2016-11-15

**Authors:** Lujia Chen, Zhongxi Huang, Guangyu Yao, Xiaoming Lyu, Jinbang Li, Xiaolei Hu, Yahong Cai, Wenji Li, Changsheng Ye, Xin Li

**Affiliations:** 1 Breast Center, Nanfang Hospital, Southern Medical University, Guangzhou, 510515 Guangdong People’s Republic of China; 2 Cancer Research Institute and the Provincial Key Laboratory of Functional Proteomics, Southern Medical University, Guangzhou, 510515 Guangdong People’s Republic of China; 3 Department of Laboratory Medicine, The Third Affiliated Hospital, Southern Medical University, Guangzhou, 510630 Guangdong People’s Republic of China

## Erratum to: Journal of Translational Medicine (2015) 13:168 DOI 10.1186/s12967-015-0521-1

Unfortunately, the original version of this article [1] contained an error. The sequence of the author names was incorrect. The corrected order of the author list can be found in this erratum.
