# Supplementary material for: The expression of CXCL13 and its relation to unfavorable clinical characteristics in young breast cancer
Source: J Transl Med. 2015 May 20;13:168. doi: 10.1186/s12967-015-0521-1 (PMC4471911; doi:10.1186/s12967-015-0521-1)
Supplement: Additional file 1: Table S1. — Clinical characteristic of gene microarray with breast cancer. [file 12967_2015_521_MOESM1_ESM.doc]

**Supplementary Table S1 Clinical Characteristic of Gene Microarray with Breast Cancer**

| Characteristic | GSE45255 | | | GSE15852 | | |
| --- | --- | --- | --- | --- | --- | --- |
|  | ≤45 ys 45-65 ys ≥65 ys  (n=23) (n=55) (n=17)  No. % No. % No. % | | | ≤45 ys 45-65 ys ≥65 ys  (n=13) (n=26) (n=4)  No. % No. % No. % | | |
| Age, years  Range  Median  Race  Chinese  Indian  Malay  Tumor size, mm  Range  Median  Tumor grade  1  2  3  Lymph node status  Positive  Negative  ER status  Positive  Negative  Missing  PR status  Positive  Negative  Missing  HER2 status  Positive  Negative  Missing | 29-45  41  ---  ---  ---  15-80  25  3 13.1  7 30.4  13 56.5  10 43.5  13 56.5  15 65.2  8 34.8  --- ---  14 60.9  9 39.1  --- ---  10 43.5  12 52.2  1 4.3 | 46-64  52  ---  ---  ---  12-60  25  4 7.3  22 40.0  29 52.7  28 50.9  27 49.1  31 56.4  23 41.8  1 1.8  29 52.7  25 45.5  1 1.8  29 52.7  24 43.6  2 3.7 | 65-86  73  ---  ---  ---  17-40  30  4 23.5  9 53.0  4 23.5  7 41.2  10 58.8  15 88.2  2 11.8  --- ---  12 70.6  5 29.4  --- ---  7 41.2  10 58.8  --- --- | 22-45  41  2 15.4  1 7.7  10 76.9  ---  ---  2 15.4  6 46.1  5 38.5  --- ---  --- ---  --- ---  --- ---  --- ---  --- ---  --- ---  --- ---  --- ---  --- ---  --- --- | 46-64  53.5  7 27.0  3 11.5  16 61.5  ---  ---  6 23.1  14 53.8  6 23.1  --- ---  --- ---  --- ---  --- ---  --- ---  --- ---  --- ---  --- ---  --- ---  --- ---  --- --- | 65-79  72.5  1 25.0  --- ---  3 75.0  ---  ---  --- ---  3 75.0  1 25.0  --- ---  --- ---  --- ---  --- ---  --- ---  --- ---  --- ---  --- ---  --- ---  --- ---  --- --- |
