# Supplementary material for: The expression of CXCL13 and its relation to unfavorable clinical characteristics in young breast cancer
Source: J Transl Med. 2015 May 20;13:168. doi: 10.1186/s12967-015-0521-1 (PMC4471911; doi:10.1186/s12967-015-0521-1)
Supplement: Additional file 2: Table S2. — 553 differentially expressed genes identified by SAM Method. [file 12967_2015_521_MOESM2_ESM.doc]

**Supplementary Table S2 553 Differentially Expressed Genes Identified by SAM Method**

| NO. | Gene_symbol | Gene_name | Fold change | P value |
| --- | --- | --- | --- | --- |
| 1  2  3  4  5  6  7  8  9  10  11  12  13  14  15  16  17  18  19  20  21  22 | KRT14  GABRP  PROM1  CXCL13  IGHM  IGLL3P  MMP7  IGJ  KRT17  GUSBP11  KRT15  KRT7  TSPYL5  IGKC  S100A2  LDHB  SFRP1  KRT5  CDH3  RND3  SYNM  KIT | Keratin 14  Gamma-aminobutyric acid (GABA) A receptor, pi  Prominin 1  Chemokine (C-X-C motif) ligand 13  Immunoglobulin heavy constant mu  Immunoglobulin lambda-like polypeptide 3, pseudogene  Matrix metallopeptidase 7 (matriysin, uterine)  Immunoglobulin J polypeptide, linker protein for immunoglobulin alpha and mu polypeptides  Keratin 17  Glucuronidase, beta pseudogene 11  Keratin 15  Keratin 7  TSPY-like 5  Immunoglobulin kappa constant  S100 calcium binding protein A2  Lactate dehydrogenase B  Secreted frizzled-related protein 1  Keratin 5  Cadherin 3, type 1, P-cadherin (placental)  Rho family GTPase 3  Synemin, intermediate filament protein  V-kit Hardy-Zuckerman 4 feline sarcoma viral oncogene homolog | 4.31  2.82  2.76  2.64  2.63  2.61  2.52  2.40  2.37  2.35  2.16  2.14  2.03  1.97  1.91  1.85  1.83  1.79  1.78  1.78  1.76  1.75 | 5.8×10^-7^  7.2×10^-6^  2.6×10^-4^  8.2×10^-4^  1.7×10^-4^  1.5×10^-3^  2.3×10^-4^  8.0×10^-4^  7.4×10^-7^  2.0×10^-3^  1.4×10^-3^  3.1×10^-3^  5.0×10^-5^  2.4×10^-3^  2.1×10^-4^  6.4×10^-4^  5.5×10^-4^  6.1×10^-4^  9.9×10^-6^  9.7×10^-6^  1.8×10^-4^  7.0×10^-6^ |

(To continue)

| NO. | Gene_symbol | Gene_name | Fold change | P value |
| --- | --- | --- | --- | --- |
| 23  24  25  26  27  28  29  30  31  32  33  34  35  36  37  38  39  40  41  42  43  44  45  46  47 | KRT6B  MT1X  AMIGO2  SLC6A14  RGS2  SRGN  DDIT4  NFIB  DTX4  CSDA  GBP2  ITM2A  PADI2  RBMS1  SFN  PLAGL1  DLK1  PLAC8  NT5DC2  NDRG1  NXN  PLCB4  FAT1  BTBD3  BBOX1 | Keratin 6B  Metallothionein 1X  Adhesion molecule with Ig-like domain  Solute carrier family 6 (amino acid transporter), member 14  Regulator of G-protein signaling 2, 24kDa  Serglycin  DNA-damage-inducible transcript 4  Nuclear factor I/B  Deltex homolog 4 (Drosophila)  Cold shock domain protein A  Guanylate binding protein 2, interferon-inducible  Integral membrane protein 2A  Peptidyl arginine deiminase, type II  RNA binding motif, single stranded interacting protein 1  Stratifin  Pleiomorphic adenoma gene-like 1  Delta-like 1 homolog (Drosophila)  Placenta-specific 8  5'-Nucleotidase domain containing 2  N-myc downstream regulated 1  Nucleoredoxin  Phospholipase C, beta 4  FAT tumor suppressor homolog 1 (Drosophila)  BTB (POZ) domain containing 3  Butyrobetaine (gamma), 2-oxoglutarate dioxygenase (gamma-butyrobetaine hydroxylase) 1 | 1.75  1.74  1.65  1.63  1.61  1.60  1.60  1.58  1.58  1.57  1.54  1.53  1.52  1.52  1.49  1.48  1.47  1.47  1.45  1.45  1.44  1.43  1.41  1.41  1.41 | 1.3×10^-4^  1.8×10^-3^  2.1×10^-3^  4.3×10^-4^  9.3×10^-4^  2.9×10^-3^  1.4×10^-3^  1.8×10^-3^  1.4×10^-4^  3.8×10^-4^  1.8×10^-3^  1.9×10^-3^  1.4×10^-4^  3.2×10^-4^  7.1×10^-5^  1.8×10^-3^  1.3×10^-3^  1.4×10^-4^  2.4×10^-3^  2.7×10^-3^  2.0×10^-4^  5.7×10^-4^  1.7×10^-3^  2.6×10^-4^  3.9×10^-4^ |

(To continue)

| NO. | Gene_symbol | Gene_name | Fold change | P value |
| --- | --- | --- | --- | --- |
| 48  49  50  51  52  53  54  55  56  57  58  59  60  61  62  63  66  65  66  67  68  69  70  71  72 | ACTG2  ID4  SCPEP1  CISD1  TRIM29  ROBO1  ARL4C  LBR  MID1  RUNX3  RAP2A  FMO1  PTDSS1  ADSL  MPHOSPH6  TES  MEX3C  CRLF3  POLR2F  KLF5  TNFRSF17  MTRR  CHN1  KIAA0020  PAPD7 | Actin, gamma 2, smooth muscle, enteric  Inhibitor of DNA binding 4, dominant negative helix-loop-helix protein  Serine carboxypeptidase 1  CDGSH iron sulfur domain 1  Tripartite motif containing 29  Roundabout, axon guidance receptor, homolog 1 (Drosophila)  ADP-ribosylation factor-like 4C  Lamin B receptor  Midline 1 (Opitz/BBB syndrome)  Runt-related transcription factor 3  RAP2A, member of RAS oncogene family  Flavin containing monooxygenase 1  Phosphatidylserine synthase 1  Adenylosuccinate lyase  M-phase phosphoprotein 6  Testis derived transcript (3 LIM domains)  Mex-3 homolog C (C. elegans)  Cytokine receptor-like factor 3  Polymerase (RNA) II (DNA directed) polypeptide F  Kruppel-like factor 5 (intestinal)  Tumor necrosis factor receptor superfamily, member 17  5-methyltetrahydrofolate-homocysteine methyltransferase reductase  Chimerin 1  KIAA0020  PAP associated domain containing 7 | 1.39  1.38  1.37  1.36  1.35  1.35  1.35  1.34  1.33  1.33  1.32  1.32  1.30  1.30  1.29  1.26  1.26  1.26  1.25  1.25  1.24  1.24  1.23  1.23  1.22 | 2.6×10^-3^  5.6×10^-4^  2.1×10^-3^  1.5×10^-3^  2.1×10^-3^  2.4×10^-4^  3.0×10^-3^  1.3×10^-3^  1.4×10^-3^  1.5×10^-4^  5.5×10^-4^  2.3×10^-4^  1.2×10^-3^  1.3×10^-5^  1.5×10^-3^  1.6×10^-3^  2.5×10^-3^  2.9×10^-3^  4.3×10^-4^  2.7×10^-3^  3.1×10^-3^  2.7×10^-3^  1.9×10^-3^  1.4×10^-3^  1.7×10^-3^ |

(To continue)

| NO. | Gene_symbol | Gene_name | Fold change | P value |
| --- | --- | --- | --- | --- |
| 73  74  75  76  77  78  79  80  81  82  83  84  85  86  87  88  89  90  91  92  93  94  95  96  97 | EML4  RLF  LPIN1  SNRPD1  RNASEH1  ZNF259  EHBP1  SNX5  MTM1  RASA3  APC  KCNH6  AAR2  PADI4  HOXD12  C10orf76  TREH  YIPF2  LRP5L  ARHGAP33  ATRN  ZNF764  BNIP1  POFUT2  ZAK | Echinoderm microtubule associated protein like 4  Rearranged L-myc fusion  Lipin 1  Small nuclear ribonucleoprotein D1 polypeptide 16kDa  Ribonuclease H1  Zinc finger protein 259  EH domain binding protein 1  Sorting nexin 5  Myotubularin 1  RAS p21 protein activator 3  Adenomatous polyposis coli  Potassium voltage-gated channel, subfamily H (eag-related), member 6  AAR2 splicing factor homolog (S. cerevisiae)  Peptidyl arginine deiminase, type IV  Homeobox D12  Chromosome 10 open reading frame 76  Trehalase (brush-border membrane glycoprotein)  Yip1 domain family, member 2  Low density lipoprotein receptor-related protein 5-like  Rho GTPase activating protein 33  Attractin  Zinc finger protein 764  BCL2/adenovirus E1B 19kDa interacting protein 1  Protein O-fucosyltransferase 2  Sterile alpha motif and leucine zipper containing kinase AZK | 1.20  1.19  1.19  1.18  1.17  1.17  1.12  1.12  0.96  0.95  0.94  0.94  0.94  0.93  0.93  0.93  0.93  0.93  0.93  0.93  0.93  0.93  0.93  0.93  0.93 | 2.2×10^-3^  1.4×10^-3^  2.3×10^-3^  7.6×10^-4^  5.7×10^-5^  4.1×10^-4^  1.2×10^-3^  2.6×10^-3^  1.6×10^-2^  2.0×10^-2^  2.5×10^-2^  2.6×10^-2^  1.9×10^-2^  1.4×10^-2^  1.1×10^-2^  9.8×10^-3^  1.4×10^-2^  2.1×10^-2^  1.3×10^-2^  2.5×10^-2^  2.4×10^-2^  2.7×10^-2^  1.8×10^-2^  2.4×10^-2^  2.0×10^-2^ |

(To continue)

| NO. | Gene_symbol | Gene_name | Fold change | P value |
| --- | --- | --- | --- | --- |
| 98  99  100  101  102  103  104  105  106  107  108  109  110  111  112  113  114  115  116  117  118  119  120  121  122 | RREB1  XAB2  RAPGEF4  TJAP1  ABHD8  ZNF668  BAIAP2  ST8SIA2  ST7  ORAI2  ACBD4  SLC35F6  CDKL3  MXD1  NDOR1  ATP9B  CYP20A1  KLHL36  CASZ1  ZNF174  ATOH1  RHOT2  NUDT6  SSTR4  VAMP4 | Ras responsive element binding protein 1  XPA binding protein 2  Rap guanine nucleotide exchange factor (GEF) 4  Tight junction associated protein 1 (peripheral)  Abhydrolase domain containing 8  Zinc finger protein 668  BAI1-associated protein 2  ST8 alpha-N-acetyl-neuraminide alpha-2,8-sialyltransferase 2  Suppression of tumorigenicity 7  ORAI calcium release-activated calcium modulator 2  Acyl-CoA binding domain containing 4  Solute carrier family 35, member F6  Cyclin-dependent kinase-like 3  MAX dimerization protein 1  NADPH dependent diflavin oxidoreductase 1  ATPase, class II, type 9B  Cytochrome P450, family 20, subfamily A, polypeptide 1  Kelch-like family member 36  Castor zinc finger 1  Zinc finger protein 174  Atonal homolog 1 (Drosophila)  Ras homolog family member T2  Nudix (nucleoside diphosphate linked moiety X)-type motif 6  Somatostatin receptor 4  Vesicle-associated membrane protein 4 | 0.92  0.92  0.92  0.92  0.92  0.92  0.92  0.92  0.92  0.92  0.92  0.92  0.92  0.92  0.92  0.92  0.92  0.92  0.92  0.92  0.92  0.92  0.92  0.92  0.92 | 9.0×10^-3^  2.6×10^-2^  2.8×10^-3^  6.8×10^-3^  5.8×10^-3^  9.4×10^-3^  2.0×10^-2^  1.7×10^-2^  1.5×10^-2^  2.3×10^-2^  1.1×10^-2^  9.8×10^-3^  1.5×10^-2^  2.5×10^-2^  2.6×10^-2^  2.1×10^-2^  1.5×10^-2^  1.8×10^-2^  2.0×10^-2^  3.0×10^-3^  2.2×10^-2^  2.1×10^-2^  5.0×10^-3^  1.3×10^-2^  2.6×10^-2^ |

(To continue)

| NO. | Gene_symbol | Gene_name | Fold change | P value |
| --- | --- | --- | --- | --- |
| 123  124  125  126  127  128  129  130  131  132  133  134  135  136  137  138  139  140  141  142  143  144  145  146  147 | TAOK2  ZNF839  MAPK8IP3  NAA60  GPN2  RABEPK  PSMC3IP  MLH3  COQ7  SMPD1  GEMIN8  ZNF557  SLC48A1  GPR37L1  C2CD2L  PSEN2  NARFL  CTRC  RAB40C  CRYBA4  USP20  SNX19  CHRND  SPATA6  PKD1 | TAO kinase 2  Zinc finger protein 839  Mitogen-activated protein kinase 8 interacting protein 3  N(alpha)-acetyltransferase 60, NatF catalytic subunit  GPN-loop GTPase 2  Rab9 effector protein with kelch motifs  PSMC3 interacting protein  MutL homolog 3 (E. coli)  Coenzyme Q7 homolog, ubiquinone (yeast)  Sphingomyelin phosphodiesterase 1, acid lysosomal  Gem (nuclear organelle) associated protein 8  Zinc finger protein 557  Solute carrier family 48 (heme transporter), member 1  G protein-coupled receptor 37 like 1  C2CD2-like  Presenilin 2 (Alzheimer disease 4)  Nuclear prelamin A recognition factor-like  Chymotrypsin C (caldecrin)  RAB40C, member RAS oncogene family  Crystallin, beta A4  Ubiquitin specific peptidase 20  Sorting nexin 19  Cholinergic receptor, nicotinic, delta (muscle)  Spermatogenesis associated 6  Polycystic kidney disease 1 (autosomal dominant) | 0.92  0.92  0.92  0.92  0.91  0.91  0.91  0.91  0.91  0.91  0.91  0.91  0.91  0.91  0.91  0.91  0.91  0.91  0.91  0.91  0.91  0.91  0.91  0.91  0.91 | 1.5×10^-3^  1.9×10^-2^  2.1×10^-2^  1.4×10^-2^  1.1×10^-2^  2.0×10^-2^  1.5×10^-2^  1.4×10^-2^  1.2×10^-2^  1.7×10^-2^  1.4×10^-2^  1.8×10^-2^  1.3×10^-2^  1.0×10^-2^  6.5×10^-3^  1.3×10^-2^  2.4×10^-2^  2.2×10^-2^  2.4×10^-2^  2.3×10^-2^  2.0×10^-2^  6.5×10^-3^  2.4×10^-2^  1.8×10^-2^  2.1×10^-2^ |

(To continue)

| NO. | Gene_symbol | Gene_name | Fold change | P value |
| --- | --- | --- | --- | --- |
| 148  149  150  151  152  153  154  155  156  157  158  159  160  161  162  163  164  165  166  167  168  169  170  171  172 | ITPK1  SAGE1  RNF40  GTF2F1  LANCL1  PARP3  RABL5  UBXN6  HLCS  C19orf24  VILL  AHI1  KIAA0141  CAMK1  PRKAG2  NCOR1  INPP5E  KANSL3  PROC  AZI2  SMAD3  SH2B1  PLEKHH3  ZNF219  CEP63 | Inositol-tetrakisphosphate 1-kinase  Sarcoma antigen 1  Ring finger protein 40, E3 ubiquitin protein ligase  General transcription factor IIF, polypeptide 1, 74kDa  LanC lantibiotic synthetase component C-like 1 (bacterial)  Poly (ADP-ribose) polymerase family, member 3  RAB, member RAS oncogene family-like 5  UBX domain protein 6  Holocarboxylase synthetase (biotin-(proprionyl-CoA-carboxylase (ATP-hydrolysing)) ligase)  Chromosome 19 open reading frame 24  Villin-like  Abelson helper integration site 1  KIAA0141  Calcium/calmodulin-dependent protein kinase I  Protein kinase, AMP-activated, gamma 2 non-catalytic subunit  Nuclear receptor corepressor 1  Inositol polyphosphate-5-phosphatase, 72 kDa  KAT8 regulatory NSL complex subunit 3  Protein C (inactivator of coagulation factors Va and VIIIa)  5-Azacytidine induced 2  SMAD family member 3  SH2B adaptor protein 1  Pleckstrin homology domain containing, family H (with MyTH4 domain) member 3  Zinc finger protein 219  Centrosomal protein 63kDa | 0.91  0.91  0.90  0.90  0.90  0.90  0.90  0.90  0.90  0.90  0.90  0.90  0.90  0.90  0.90  0.90  0.90  0.90  0.90  0.90  0.90  0.90  0.90  0.90  0.90 | 8.7×10^-3^  1.4×10^-2^  2.2×10^-2^  2.2×10^-2^  8.1×10^-3^  2.4×10^-2^  1.7×10^-2^  2.5×10^-2^  4.0×10^-3^  1.9×10^-2^  2.6×10^-2^  9.3×10^-3^  1.9×10^-2^  2.6×10^-2^  2.0×10^-2^  2.3×10^-2^  2.5×10^-2^  2.4×10^-2^  1.6×10^-2^  2.4×10^-2^  6.0×10^-3^  5.2×10^-3^  2.2×10^-4^  9.4×10^-3^  7.6×10^-3^ |

(To continue)

| NO. | Gene_symbol | Gene_name | Fold change | P value |
| --- | --- | --- | --- | --- |
| 173  174  175  176  177  178  179  180  181  182  183  184  185  186  187  188  189  190  191  192  193  194  195  196  197 | PTGER3  HMGCL  WDR48  SIN3B  PRRG2  LTB4R  DFNB31  CNOT3  AAGAB  CYP11B1  ZNF75D  TBC1D9B  TYW1  HIRIP3  CDK9  KIAA0753  C12orf52  TMEM8B  B4GALT7  LPPR2  DKK2  MYH10  EFCAB2  AMDHD2  ACAN | Prostaglandin E receptor 3 (subtype EP3)  3-Hydroxymethyl-3-methylglutaryl-CoA lyase  WD repeat domain 48  SIN3 transcription regulator homolog B (yeast)  Proline rich Gla (G-carboxyglutamic acid) 2  Leukotriene B4 receptor  Deafness, autosomal recessive 31  CCR4-NOT transcription complex, subunit 3  Alpha- and gamma-adaptin binding protein  Cytochrome P450, family 11, subfamily B, polypeptide 1  Zinc finger protein 75D  TBC1 domain family, member 9B (with GRAM domain)  tRNA-yW synthesizing protein 1 homolog (S. cerevisiae)  HIRA interacting protein 3  Cyclin-dependent kinase 9  KIAA0753  Chromosome 12 open reading frame 52  Transmembrane protein 8B  Xylosylprotein beta 1,4-galactosyltransferase, polypeptide 7  Lipid phosphate phosphatase-related protein type 2  Dickkopf 2 homolog (Xenopus laevis)  Myosin, heavy chain 10, non-muscle  EF-hand calcium binding domain 2  Amidohydrolase domain containing 2  Aggrecan | 0.90  0.90  0.90  0.90  0.90  0.90  0.90  0.90  0.90  0.90  0.90  0.90  0.90  0.90  0.90  0.90  0.89  0.89  0.89  0.89  0.89  0.89  0.89  0.89  0.89 | 2.4×10^-2^  1.9×10^-2^  6.1×10^-3^  7.3×10^-3^  2.0×10^-2^  8.8×10^-3^  2.6×10^-2^  2.3×10^-2^  9.8×10^-3^  8.0×10^-3^  1.8×10^-3^  4.0×10^-3^  2.6×10^-2^  3.6×10^-4^  1.2×10^-2^  2.3×10^-2^  6.6×10^-3^  1.1×10^-2^  8.1×10^-3^  1.4×10^-3^  2.1×10^-2^  1.4×10^-2^  6.1×10^-3^  2.5×10^-2^  9.3×10^-4^ |

(To continue)

| NO. | Gene_symbol | Gene_name | Fold change | P value |
| --- | --- | --- | --- | --- |
| 198  199  200  201  202  203  204  205  206  207  208  209  210  211  212  213  214  215  216  217  218  219  220  221  222 | ZMYND10  ANKMY1  LRRC20  NUDT13  ECD  LMF1  TBC1D5  PRAF2  GDPD3  RABEP2  TP73-AS1  KEL  TNK2  LRRC8E  RNF2  CDK20  HIP1  C19orf40  TNFSF12  MOCS1  BBS10  TTC30A  KCNG2  HNMT  LOC81691 | Zinc finger, MYND-type containing 10  Ankyrin repeat and MYND domain containing 1  Leucine rich repeat containing 20  Nudix (nucleoside diphosphate linked moiety X)-type motif 13  Ecdysoneless homolog (Drosophila)  Lipase maturation factor 1  TBC1 domain family, member 5  PRA1 domain family, member 2  Glycerophosphodiester phosphodiesterase domain containing 3  Rabaptin, RAB GTPase binding effector protein 2  TP73 antisense RNA 1  Kell blood group, metallo-endopeptidase  Tyrosine kinase, non-receptor, 2  Leucine rich repeat containing 8 family, member E  Ring finger protein 2  Cyclin-dependent kinase 20  Huntingtin interacting protein 1  Chromosome 19 open reading frame 40  Tumor necrosis factor (ligand) superfamily, member 12  Molybdenum cofactor synthesis 1  Bardet-Biedl syndrome 10  Tetratricopeptide repeat domain 30A  Potassium voltage-gated channel, subfamily G, member 2  Histamine N-methyltransferase  Exonuclease NEF-sp | 0.89  0.89  0.89  0.89  0.89  0.89  0.89  0.89  0.89  0.89  0.89  0.89  0.89  0.89  0.89  0.89  0.89  0.89  0.89  0.89  0.89  0.89  0.89  0.89  0.89 | 2.1×10^-2^  7.6×10^-3^  2.4×10^-2^  3.0×10^-3^  2.5×10^-2^  1.2×10^-3^  2.3×10^-3^  2.4×10^-2^  2.4×10^-2^  8.6×10^-3^  5.9×10^-3^  1.4×10^-2^  9.0×10^-3^  2.4×10^-2^  2.4×10^-2^  1.7×10^-2^  6.3×10^-3^  5.3×10^-3^  1.3×10^-2^  1.1×10^-2^  1.4×10^-2^  1.1×10^-2^  2.0×10^-2^  4.1×10^-3^  7.4×10^-3^ |

(To continue)

| NO. | Gene_symbol | Gene_name | Fold change | P value |
| --- | --- | --- | --- | --- |
| 223  224  225  226  227  228  229  230  231  232  233  234  235  236  237  238  239  240  241  242  243  244  245  246  247 | TUBG2  THTPA  COL5A3  VWA8  ZNF446  N4BP3  BCKDK  AGXT2L1  TRPC7  TSPAN31  BCAM  RABL6  ADCK2  NCSTN  MANBA  GOLGA1  GORASP1  AGPAT2  SUOX  IFT46  CHD3  BCL7B  KCNK12  SLC25A12  PTPN18 | Tubulin, gamma 2  Thiamine triphosphatase  Collagen, type V, alpha 3  Von Willebrand factor A domain containing 8  Zinc finger protein 446  NEDD4 binding protein 3  Branched chain ketoacid dehydrogenase kinase  Alanine-glyoxylate aminotransferase 2-like 1  Transient receptor potential cation channel, subfamily C, member 7  Tetraspanin 31  Basal cell adhesion molecule (Lutheran blood group)  RAB, member RAS oncogene family-like 6  AarF domain containing kinase 2  Nicastrin  Mannosidase, beta A, lysosomal  Golgin A1  Golgi reassembly stacking protein 1, 65kDa  1-Acylglycerol-3-phosphate O-acyltransferase 2  Sulfite oxidase  Intraflagellar transport 46 homolog (Chlamydomonas)  Chromodomain helicase DNA binding protein 3  B-cell CLL/lymphoma 7B  Potassium channel, subfamily K, member 12  Solute carrier family 25 (aspartate/glutamate carrier), member 12  Protein tyrosine phosphatase, non-receptor type 18 (brain-derived) | 0.89  0.89  0.89  0.89  0.89  0.88  0.88  0.88  0.88  0.88  0.88  0.88  0.88  0.88  0.88  0.88  0.88  0.88  0.88  0.88  0.88  0.88  0.88  0.88  0.88 | 1.8×10^-2^  1.3×10^-2^  8.5×10^-3^  4.4×10^-3^  2.4×10^-3^  1.4×10^-2^  2.2×10^-2^  4.4×10^-3^  2.1×10^-2^  4.3×10^-3^  1.9×10^-2^  2.5×10^-2^  1.2×10^-5^  9.6×10^-3^  5.1×10^-3^  1.8×10^-3^  1.1×10^-2^  7.9×10^-3^  1.8×10^-2^  4.2×10^-3^  1.5×10^-3^  3.3×10^-3^  7.7×10^-4^  2.7×10^-2^  6.1×10^-3^ |

(To continue)

| NO. | Gene_symbol | Gene_name | Fold change | P value |
| --- | --- | --- | --- | --- |
| 248  249  250  251  252  253  254  255  256  257  258  259  260  261  262  263  264  265  266  267  268  269  270  271  272 | PCSK6  AP4M1  STK16  IPO9  RAD50  GTF3C1  PEX19  PDE4A  DENND2A  LOC155060  TIMM22  HSDL2  LOC100506190  ICA1  SLC9A1  COPZ2  GLYR1  EPOR  CRTAP  ANO10  PALM  POMGNT1  CXXC4  SH2D4A  COQ10B | Proprotein convertase subtilisin/kexin type 6  Adaptor-related protein complex 4, mu 1 subunit  Serine/threonine kinase 16  Importin 9  RAD50 homolog (S. cerevisiae)  General transcription factor IIIC, polypeptide 1, alpha 220kDa  Peroxisomal biogenesis factor 19  Phosphodiesterase 4A, cAMP-specific  DENN/MADD domain containing 2A  AI894139 pseudogene  Translocase of inner mitochondrial membrane 22 homolog (yeast)  Hydroxysteroid dehydrogenase like 2  Uncharacterized LOC100506190  Islet cell autoantigen 1, 69kDa  Solute carrier family 9, subfamily A (NHE1, cation proton antiporter 1), member 1  Coatomer protein complex, subunit zeta 2  Glyoxylate reductase 1 homolog (Arabidopsis)  Erythropoietin receptor  Cartilage associated protein  Anoctamin 10  Paralemmin  Protein O-linked mannose beta1,2-N-acetylglucosaminyltransferase  CXXC finger protein 4  SH2 domain containing 4A  Coenzyme Q10 homolog B (S. cerevisiae) | 0.88  0.88  0.88  0.88  0.88  0.88  0.88  0.88  0.88  0.88  0.88  0.88  0.88  0.88  0.88  0.88  0.88  0.88  0.87  0.87  0.87  0.87  0.87  0.87  0.87 | 1.4×10^-2^  1.8×10^-2^  4.0×10^-3^  2.0×10^-2^  9.8×10^-3^  5.5×10^-3^  8.3×10^-3^  8.5×10^-3^  1.2×10^-2^  2.5×10^-2^  6.6×10^-3^  1.4×10^-2^  9.8×10^-3^  6.6×10^-3^  8.0×10^-3^  2.6×10^-2^  1.7×10^-2^  4.5×10^-3^  2.1×10^-3^  1.5×10^-2^  2.2×10^-2^  6.2×10^-3^  8.1×10^-3^  1.3×10^-2^  2.1×10^-2^ |

(To continue)

| NO. | Gene_symbol | Gene_name | Fold change | P value |
| --- | --- | --- | --- | --- |
| 273  274  275  276  277  278  279  280  281  282  283  284  285  286  287  288  289  290  291  292  293  294  295  296  297 | PARN  SMARCC2  LYRM9  POMT1  SUGP2  RALGPS1  CORO2A  APBB3  VIPR2  ENDOG  ORAI3  FRY  TSNAXIP1  ABHD14A  WDR52  DZIP3  LRRC48  ROGDI  FRAT1  PHF15  ACSM5  TBC1D17  COQ6  NOVA1  THAP10 | Poly(A)-specific ribonuclease  SWI/SNF related, matrix associated, actin dependent regulator of chromatin, subfamily c, member 2  LYR motif containing 9  Protein-O-mannosyltransferase 1  SURP and G patch domain containing 2  Ral GEF with PH domain and SH3 binding motif 1  Coronin, actin binding protein, 2A  Amyloid beta (A4) precursor protein-binding, family B, member 3  Vasoactive intestinal peptide receptor 2  Endonuclease G  ORAI calcium release-activated calcium modulator 3  Furry homolog (Drosophila)  Translin-associated factor X interacting protein 1  Abhydrolase domain containing 14A  WD repeat domain 52  DAZ interacting protein 3, zinc finger  Leucine rich repeat containing 48  Rogdi homolog (Drosophila)  Frequently rearranged in advanced T-cell lymphomas  PHD finger protein 15  Acyl-CoA synthetase medium-chain family member 5  TBC1 domain family, member 17  Coenzyme Q6 homolog, monooxygenase (S. cerevisiae)  Neuro-oncological ventral antigen 1  THAP domain containing 10 | 0.87  0.87  0.87  0.87  0.87  0.87  0.87  0.87  0.87  0.87  0.87  0.87  0.87  0.87  0.87  0.87  0.87  0.87  0.87  0.87  0.87  0.87  0.87  0.87  0.87 | 1.6×10^-2^  9.7×10^-5^  1.4×10^-2^  2.2×10^-2^  5.6×10^-3^  9.5×10^-5^  2.6×10^-2^  1.2×10^-3^  3.0×10^-3^  6.7×10^-3^  7.4×10^-3^  2.6×10^-2^  4.4×10^-3^  2.3×10^-2^  2.4×10^-3^  6.5×10^-3^  8.4×10^-3^  2.3×10^-2^  5.8×10^-3^  4.4×10^-4^  1.4×10^-2^  1.1×10^-3^  1.8×10^-2^  2.0×10^-2^  2.6×10^-2^ |

(To continue)

| NO. | Gene_symbol | Gene_name | Fold change | P value |
| --- | --- | --- | --- | --- |
| 298  299  300  301  302  303  304  305  306  307  308  309  310  311  312  313  314  315  316  317  318  319  320  321  322 | G6PC3  APOBR  MAPK9  FIG4  ITIH4  BTD  MR1  C16orf62  RASL11B  TSPAN15  CELSR1  COG2  PPEF1  CASP6  BCL2  DPM2  NAGLU  PGPEP1  CTDSPL  PAWR  TJP3  VIPR1  CHRD  CCDC15  RMND5B | Glucose 6 phosphatase, catalytic, 3  Apolipoprotein B receptor  Mitogen-activated protein kinase 9  FIG4 homolog, SAC1 lipid phosphatase domain containing (S. cerevisiae)  Inter-alpha-trypsin inhibitor heavy chain family, member 4  Biotinidase  Major histocompatibility complex, class I-related  Chromosome 16 open reading frame 62  RAS-like, family 11, member B  Tetraspanin 15  Cadherin, EGF LAG seven-pass G-type receptor 1  Component of oligomeric golgi complex 2  Protein phosphatase, EF-hand calcium binding domain 1  Caspase 6, apoptosis-related cysteine peptidase  B-cell CLL/lymphoma 2  Dolichyl-phosphate mannosyltransferase polypeptide 2, regulatory subunit  N-acetylglucosaminidase, alpha  Pyroglutamyl-peptidase I  CTD (carboxy-terminal domain, RNA polymerase II, polypeptide A) small phosphatase-like  PRKC, apoptosis, WT1, regulator  Tight junction protein 3  Vasoactive intestinal peptide receptor 1  Chordin  Coiled-coil domain containing 15  Required for meiotic nuclear division 5 homolog B (S. cerevisiae) | 0.87  0.87  0.87  0.87  0.86  0.86  0.86  0.86  0.86  0.86  0.86  0.86  0.86  0.86  0.86  0.86  0.86  0.86  0.86  0.86  0.86  0.86  0.86  0.86  0.86 | 3.0×10^-4^  1.6×10^-2^  7.3×10^-3^  1.8×10^-2^  6.2×10^-3^  4.4×10^-3^  1.9×10^-3^  2.4×10^-2^  1.0×10^-2^  4.6×10^-3^  6.5×10^-3^  2.4×10^-2^  1.9×10^-2^  2.2×10^-2^  2.5×10^-3^  3.1×10^-3^  6.5×10^-3^  1.8×10^-2^  9.8×10^-3^  2.0×10^-2^  5.1×10^-3^  1.6×10^-2^  2.2×10^-2^  1.8×10^-2^  1.9×10^-2^ |

(To continue)

| NO. | Gene_symbol | Gene_name | Fold change | P value |
| --- | --- | --- | --- | --- |
| 323  324  325  326  327  328  329  330  331  332  333  334  335  336  337  338  339  340  341  342  343  344  345  346  347 | CNNM3  SPEF1  FAF2  KEAP1  NUDT18  WWOX  KIAA1279  TENM4  YIPF3  MSX1  INTS12  TPCN1  HHLA3  PRND  GATAD1  DUOX1  TELO2  FHIT  PIGO  PCYOX1L  BBS4  IPP  SORBS3  NEK11  CACNA1H | Cyclin M3  Sperm flagellar 1  Fas associated factor family member 2  Kelch-like ECH-associated protein 1  Nudix (nucleoside diphosphate linked moiety X)-type motif 18  WW domain containing oxidoreductase  KIAA1279  Teneurin transmembrane protein 4  Yip1 domain family, member 3  Msh homeobox 1  Integrator complex subunit 12  Two pore segment channel 1  HERV-H LTR-associating 3  Prion protein 2 (dublet)  GATA zinc finger domain containing 1  Dual oxidase 1  TEL2, telomere maintenance 2, homolog (S. cerevisiae)  Fragile histidine triad  Phosphatidylinositol glycan anchor biosynthesis, class O  Prenylcysteine oxidase 1 like  Bardet-Biedl syndrome 4  Intracisternal A particle-promoted polypeptide  Sorbin and SH3 domain containing 3  NIMA-related kinase 11  Calcium channel, voltage-dependent, T type, alpha 1H subunit | 0.86  0.86  0.86  0.86  0.86  0.86  0.86  0.86  0.86  0.86  0.86  0.86  0.85  0.85  0.85  0.85  0.85  0.85  0.85  0.85  0.85  0.85  0.85  0.85  0.85 | 6.5×10^-3^  7.1×10^-4^  9.4×10^-4^  1.7×10^-2^  3.6×10^-3^  2.4×10^-2^  1.3×10^-2^  1.8×10^-2^  1.1×10^-3^  1.9×10^-2^  2.4×10^-2^  3.9×10^-3^  2.5×10^-2^  5.3×10^-3^  1.2×10^-2^  7.6×10^-3^  3.5×10^-3^  1.9×10^-2^  2.6×10^-3^  1.6×10^-2^  4.0×10^-3^  2.3×10^-2^  1.9×10^-3^  5.2×10^-3^  4.3×10^-3^ |

(To continue)

| NO. | Gene_symbol | Gene_name | Fold change | P value |
| --- | --- | --- | --- | --- |
| 348  349  350  351  352  353  354  355  356  357  358  359  360  361  362  363  364  365  366  367  368  369  370  371  372 | KLHL26  SLC29A3  SYNC  SLC1A2  CREB3  MEGF9  NIT1  KIAA0556  TMEM120B  TLE3  TRAK1  TENC1  MYOZ3  C16orf58  PROP1  TNIK  CNNM4  CYB561D2  FLJ22184  UBXN8  NOL3  TMEM5  RTN2  IQCK  DGKD | Kelch-like family member 26  Solute carrier family 29 (nucleoside transporters), member 3  Syncoilin, intermediate filament protein  Solute carrier family 1 (glial high affinity glutamate transporter), member 2  cAMP responsive element binding protein 3  Multiple EGF-like-domains 9  Nitrilase 1  KIAA0556  Transmembrane protein 120B  Transducin-like enhancer of split 3 (E(sp1) homolog, Drosophila)  Trafficking protein, kinesin binding 1  Tensin like C1 domain containing phosphatase (tensin 2)  Myozenin 3  Chromosome 16 open reading frame 58  PROP paired-like homeobox 1  TRAF2 and NCK interacting kinase  Cyclin M4  Cytochrome b-561 domain containing 2  Putative uncharacterized protein FLJ22184  UBX domain protein 8  Nucleolar protein 3 (apoptosis repressor with CARD domain)  Transmembrane protein 5  Reticulon 2  IQ motif containing K  Diacylglycerol kinase, delta 130kDa | 0.85  0.85  0.85  0.85  0.85  0.85  0.85  0.85  0.85  0.85  0.85  0.85  0.85  0.84  0.84  0.84  0.84  0.84  0.84  0.84  0.84  0.84  0.84  0.84  0.84 | 1.6×10^-4^  1.5×10^-2^  2.5×10^-3^  1.5×10^-2^  2.2×10^-2^  2.7×10^-2^  9.0×10^-4^  9.2×10^-3^  3.4×10^-3^  1.4×10^-3^  1.1×10^-3^  6.9×10^-3^  9.0×10^-3^  5.3×10^-4^  1.6×10^-2^  1.8×10^-2^  6.5×10^-3^  4.1×10^-3^  1.6×10^-2^  1.4×10^-2^  8.4×10^-3^  5.1×10^-3^  2.4×10^-3^  7.5×10^-3^  1.2×10^-2^ |

(To continue)

| NO. | Gene_symbol | Gene_name | Fold change | P value |
| --- | --- | --- | --- | --- |
| 373  374  375  376  377  378  379  380  381  382  383  384  385  386  387  388  389  390  391  392  393  394  395  396  397 | C9orf116  SYNJ2  ZFHX3  GLI3  SLC12A8  EPB41L1  MST1R  SALL2  C7orf63  MAN2C1  BTRC  IDUA  TMEM259  GNA14  DLX2  SMIM7  TTC12  SLC7A8  DNAAF1  GALNT10  SIL1  DOK1  FAM174B  FAM63A  MIA3 | Chromosome 9 open reading frame 116  Synaptojanin 2  Zinc finger homeobox 3  GLI family zinc finger 3  Solute carrier family 12 (potassium/chloride transporters), member 8  Erythrocyte membrane protein band 4.1-like 1  Macrophage stimulating 1 receptor (c-met-related tyrosine kinase)  Sal-like 2 (Drosophila)  Chromosome 7 open reading frame 63  Mannosidase, alpha, class 2C, member 1  Beta-transducin repeat containing E3 ubiquitin protein ligase  Iduronidase, alpha-L-  Transmembrane protein 259  Guanine nucleotide binding protein (G protein), alpha 14  Distal-less homeobox 2  Small integral membrane protein 7  Tetratricopeptide repeat domain 12  Solute carrier family 7 (amino acid transporter light chain, L system), member 8  Dynein, axonemal, assembly factor 1  UDP-N-acetyl-alpha-D-galactosamine:polypeptide N-acetylgalactosaminyltransferase 10 (GalNAc-T10)  SIL1 homolog, endoplasmic reticulum chaperone (S. cerevisiae)  Docking protein 1, 62kDa (downstream of tyrosine kinase 1)  Family with sequence similarity 174, member B  Family with sequence similarity 63, member A  Melanoma inhibitory activity family, member 3 | 0.84  0.84  0.84  0.84  0.84  0.84  0.84  0.84  0.84  0.84  0.83  0.83  0.83  0.83  0.83  0.83  0.83  0.83  0.83  0.83  0.83  0.83  0.83  0.83  0.83 | 4.7×10^-4^  8.0×10^-3^  1.8×10^-3^  2.0×10^-2^  7.5×10^-3^  4.7×10^-3^  2.4×10^-2^  1.0×10^-2^  9.3×10^-3^  6.0×10^-4^  1.1×10^-2^  2.2×10^-3^  2.0×10^-2^  5.8×10^-3^  2.1×10^-2^  9.2×10^-3^  2.3×10^-2^  8.6×10^-4^  1.1×10^-4^  2.0×10^-2^  8.3×10^-3^  8.4×10^-3^  2.3×10^-3^  1.4×10^-2^  4.1×10^-3^ |

(To continue)

| NO. | Gene_symbol | Gene_name | Fold change | P value |
| --- | --- | --- | --- | --- |
| 398  399  400  401  402  403  404  405  406  407  408  409  410  411  412  413  414  415  416  417  418  419  420  421  422 | ENPP1  RHBDF1  RAB30  MYO15B  NAPA  MZF1  STXBP2  APPL2  AMH  PPP2R5A  HHEX  ENTPD3  LGALS8  AP2B1  POLD4  RABEP1  TMEM187  THNSL2  ABLIM3  PDXDC1  MYL5  ASTN2  UTRN  P2RX4  PEX11A | Ectonucleotide pyrophosphatase/phosphodiesterase 1  Rhomboid 5 homolog 1 (Drosophila)  RAB30, member RAS oncogene family  Myosin XVB pseudogene  N-ethylmaleimide-sensitive factor attachment protein, alpha  Myeloid zinc finger 1  Syntaxin binding protein 2  Adaptor protein, phosphotyrosine interaction, PH domain and leucine zipper containing 2  Anti-Mullerian hormone  Protein phosphatase 2, regulatory subunit B', alpha  Hematopoietically expressed homeobox  Ectonucleoside triphosphate diphosphohydrolase 3  Lectin, galactoside-binding, soluble, 8  Adaptor-related protein complex 2, beta 1 subunit  Polymerase (DNA-directed), delta 4, accessory subunit  Rabaptin, RAB GTPase binding effector protein 1  Transmembrane protein 187  Threonine synthase-like 2 (S. cerevisiae)  Actin binding LIM protein family, member 3  Pyridoxal-dependent decarboxylase domain containing 1  Myosin, light chain 5, regulatory  Astrotactin 2  Utrophin  Purinergic receptor P2X, ligand-gated ion channel, 4  Peroxisomal biogenesis factor 11 alpha | 0.83  0.83  0.83  0.83  0.83  0.83  0.83  0.83  0.83  0.83  0.83  0.83  0.83  0.83  0.83  0.82  0.82  0.82  0.82  0.82  0.82  0.82  0.82  0.82  0.82 | 2.8×10^-3^  6.5×10^-3^  1.5×10^-2^  1.3×10^-2^  1.1×10^-2^  6.0×10^-3^  1.8×10^-2^  2.4×10^-2^  3.7×10^-3^  2.2×10^-4^  2.3×10^-2^  1.4×10^-2^  2.2×10^-2^  1.2×10^-2^  7.9×10^-4^  1.3×10^-2^  1.3×10^-3^  1.4×10^-3^  2.2×10^-2^  2.3×10^-2^  3.3×10^-4^  8.2×10^-3^  3.4×10^-3^  7.7×10^-3^  1.0×10^-2^ |

(To continue)

| NO. | Gene_symbol | Gene_name | Fold change | P value |
| --- | --- | --- | --- | --- |
| 423  424  425  426  427  428  429  430  431  432  433  434  435  436  437  438  439  440  441  442  443  444  445  446  447 | PLA2G12A  NEIL1  SHC2  ADCY9  TRIM45  ANG  CSRNP2  NFIC  PEX6  POLR3K  TBC1D8  PPOX  GPRC5C  ENTPD5  CERS4  OSBPL10  SYTL2  RGS11  TCTN1  OVOL2  DOPEY2  BDH1  ADORA2A  CACNA1D  PIGP | Phospholipase A2, group XIIA  Nei endonuclease VIII-like 1 (E. coli)  SHC (Src homology 2 domain containing) transforming protein 2  Adenylate cyclase 9  Tripartite motif containing 45  Angiogenin, ribonuclease, RNase A family, 5  Cysteine-serine-rich nuclear protein 2  Nuclear factor I/C (CCAAT-binding transcription factor)  Peroxisomal biogenesis factor 6  Polymerase (RNA) III (DNA directed) polypeptide K, 12.3 kDa  TBC1 domain family, member 8 (with GRAM domain)  Protoporphyrinogen oxidase  G protein-coupled receptor, family C, group 5, member C  Ectonucleoside triphosphate diphosphohydrolase 5  Ceramide synthase 4  Oxysterol binding protein-like 10  Synaptotagmin-like 2  Regulator of G-protein signaling 11  Tectonic family member 1  Ovo-like 2 (Drosophila)  Dopey family member 2  3-Hydroxybutyrate dehydrogenase, type 1  Adenosine A2a receptor  Calcium channel, voltage-dependent, L type, alpha 1D subunit  Phosphatidylinositol glycan anchor biosynthesis, class P | 0.81  0.81  0.81  0.81  0.81  0.81  0.81  0.81  0.81  0.81  0.81  0.81  0.81  0.81  0.80  0.80  0.80  0.80  0.80  0.80  0.80  0.80  0.80  0.80  0.80 | 5.3×10^-3^  3.0×10^-3^  1.5×10^-3^  2.5×10^-3^  5.7×10^-3^  2.4×10^-3^  5.9×10^-3^  2.2×10^-2^  9.4×10^-3^  2.2×10^-2^  1.7×10^-2^  1.3×10^-3^  5.3×10^-4^  1.8×10^-2^  1.5×10^-2^  2.6×10^-2^  1.3×10^-2^  2.8×10^-3^  7.2×10^-5^  7.8×10^-3^  4.3×10^-3^  1.2×10^-2^  2.7×10^-2^  1.2×10^-2^  1.6×10^-3^ |

(To continue)

| NO. | Gene_symbol | Gene_name | Fold change | P value |
| --- | --- | --- | --- | --- |
| 448  449  450  451  452  453  454  455  456  457  458  459  460  461  462  463  464  465  466  467  468  469  470  471  472 | ITPR1  ZNF43  MGAM  GP2  CBX4  FBXL7  ECI2  NAV3  ACAT1  PTPRT  UNC13B  ELMO3  ERBB4  SSH3  ALDH6A1  ECHDC3  ZNF467  CCDC176  TIMP3  P4HTM  IKBKB  RBPMS  ARHGAP32  HHAT  NR2F2 | Inositol 1,4,5-trisphosphate receptor, type 1  Zinc finger protein 43  Maltase-glucoamylase (alpha-glucosidase)  Glycoprotein 2 (zymogen granule membrane)  Chromobox homolog 4  F-box and leucine-rich repeat protein 7  Enoyl-CoA delta isomerase 2  Neuron navigator 3  Acetyl-CoA acetyltransferase 1  Protein tyrosine phosphatase, receptor type, T  Unc-13 homolog B (C. elegans)  Engulfment and cell motility 3  V-erb-a erythroblastic leukemia viral oncogene homolog 4 (avian)  Slingshot homolog 3 (Drosophila)  Aldehyde dehydrogenase 6 family, member A1  Enoyl CoA hydratase domain containing 3  Zinc finger protein 467  Coiled-coil domain containing 176  TIMP metallopeptidase inhibitor 3  Prolyl 4-hydroxylase, transmembrane (endoplasmic reticulum)  Inhibitor of kappa light polypeptide gene enhancer in B-cells, kinase beta  RNA binding protein with multiple splicing  Rho GTPase activating protein 32  Hedgehog acyltransferase  Nuclear receptor subfamily 2, group F, member 2 | 0.80  0.79  0.79  0.79  0.79  0.79  0.79  0.79  0.79  0.79  0.79  0.79  0.79  0.78  0.78  0.78  0.78  0.77  0.77  0.77  0.77  0.77  0.77  0.77  0.77 | 1.2×10^-2^  2.5×10^-3^  1.2×10^-2^  2.9×10^-3^  4.7×10^-3^  1.5×10^-2^  2.4×10^-2^  2.3×10^-2^  1.9×10^-2^  2.0×10^-2^  1.1×10^-2^  1.9×10^-2^  1.8×10^-2^  8.1×10^-4^  3.6×10^-3^  1.9×10^-2^  1.3×10^-3^  2.3×10^-2^  2.7×10^-2^  1.9×10^-2^  2.6×10^-3^  1.4×10^-2^  4.4×10^-3^  1.1×10^-3^  2.1×10^-2^ |

(To continue)

| NO. | Gene_symbol | Gene_name | Fold change | P value |
| --- | --- | --- | --- | --- |
| 473  474  475  476  477  478  479  480  481  482  483  484  485  486  487  488  489  490  491  492  493  494  495  496  497 | RAMP2  GAD1  TSC2  CYB5R1  CERS2  FMOD  FGFR2  GPC4  SPAG6  MAN1C1  PYGL  TP53TG1  EXOC2  MTL5  APBB2  TEX14  HPN  INPP4B  KDM4B  TSPAN5  COG7  KIAA1467  MANSC1  ABCA3  SCCPDH | Receptor (G protein-coupled) activity modifying protein 2  Glutamate decarboxylase 1 (brain, 67kDa)  Tuberous sclerosis 2  Cytochrome b5 reductase 1  Ceramide synthase 2  Fibromodulin  Fibroblast growth factor receptor 2  Glypican 4  Sperm associated antigen 6  Mannosidase, alpha, class 1C, member 1  Phosphorylase, glycogen, liver  TP53 target 1 (non-protein coding)  Exocyst complex component 2  Metallothionein-like 5, testis-specific (tesmin)  Amyloid beta (A4) precursor protein-binding, family B, member 2  Testis expressed 14  Hepsin  Inositol polyphosphate-4-phosphatase, type II, 105kDa  Lysine (K)-specific demethylase 4B  Tetraspanin 5  Component of oligomeric golgi complex 7  KIAA1467  MANSC domain containing 1  ATP-binding cassette, sub-family A (ABC1), member 3  Saccharopine dehydrogenase (putative) | 0.76  0.76  0.76  0.76  0.76  0.76  0.76  0.76  0.76  0.76  0.76  0.76  0.76  0.76  0.75  0.75  0.75  0.75  0.75  0.75  0.74  0.74  0.74  0.74  0.73 | 2.3×10^-4^  9.7×10^-3^  4.4×10^-3^  1.6×10^-4^  2.6×10^-2^  1.4×10^-2^  8.1×10^-3^  5.0×10^-3^  6.2×10^-3^  2.2×10^-2^  1.1×10^-2^  5.9×10^-4^  5.1×10^-3^  2.1×10^-2^  9.3×10^-5^  1.1×10^-2^  2.4×10^-2^  1.7×10^-2^  4.9×10^-3^  8.4×10^-3^  1.1×10^-4^  2.3×10^-2^  1.8×10^-2^  1.5×10^-2^  6.4×10^-3^ |

(To continue)

| NO. | Gene_symbol | Gene_name | Fold change | P value |
| --- | --- | --- | --- | --- |
| 498  499  500  501  502  503  504  505  506  507  508  509  510  511  512  513  514  515  516  517  518  519  520  521  522 | PRSS21  C16orf45  MAOA  KAL1  PIGT  DACH1  IGF1R  ZNF281  CLSTN2  REPS2  ANXA9  CCNO  FBP1  CASD1  SYBU  KIF13B  LRRC15  FMO5  SIAH2  CITED2  TCEAL1  RHOBTB3  PPP1R3C  GPRC5A  FAM134B | Protease, serine, 21 (testisin)  Chromosome 16 open reading frame 45  Monoamine oxidase A  Kallmann syndrome 1 sequence  Phosphatidylinositol glycan anchor biosynthesis, class T  Dachshund homolog 1 (Drosophila)  Insulin-like growth factor 1 receptor  Zinc finger protein 281  Calsyntenin 2  RALBP1 associated Eps domain containing 2  Annexin A9  Cyclin O  Fructose-1,6-bisphosphatase 1  CAS1 domain containing 1  Syntabulin (syntaxin-interacting)  Kinesin family member 13B  Leucine rich repeat containing 15  Flavin containing monooxygenase 5  Siah E3 ubiquitin protein ligase 2  Cbp/p300-interacting transactivator, with Glu/Asp-rich carboxy-terminal domain, 2  Transcription elongation factor A (SII)-like 1  Rho-related BTB domain containing 3  Protein phosphatase 1, regulatory subunit 3C  G protein-coupled receptor, family C, group 5, member A  Family with sequence similarity 134, member B | 0.73  0.73  0.73  0.72  0.72  0.72  0.71  0.71  0.71  0.71  0.70  0.70  0.70  0.69  0.69  0.69  0.69  0.69  0.69  0.68  0.68  0.68  0.68  0.67  0.67 | 2.9×10^-4^  6.1×10^-3^  3.5×10^-3^  2.0×10^-2^  3.8×10^-4^  4.3×10^-3^  1.6×10^-2^  1.1×10^-2^  1.7×10^-3^  4.0×10^-3^  2.1×10^-3^  5.6×10^-3^  6.4×10^-3^  1.7×10^-3^  7.2×10^-3^  1.3×10^-3^  1.6×10^-2^  1.6×10^-2^  1.9×10^-2^  4.1×10^-3^  1.2×10^-2^  5.6×10^-3^  7.1×10^-3^  7.4×10^-3^  3.6×10^-3^ |

(To continue)

| NO. | Gene_symbol | Gene_name | Fold change | P value |
| --- | --- | --- | --- | --- |
| 523  524  525  526  527  528  529  530  531  532  533  534  535  536  537  538  539  540  541  542  543  544  545  546  547 | PAXIP1  SYT17  BMPR1B  RNASE4  GLCE  PRKAR2B  SELENBP1  CCDC170  F7  AR  REEP1  SLC44A4  CLGN  PGR  PLAT  MYB  CA12  ESR1  CYP2B7P1  TBC1D9  ECM1  KCNE4  DNAJC12  SCUBE2  EVL | PAX interacting (with transcription-activation domain) protein 1  Synaptotagmin XVII  Bone morphogenetic protein receptor, type IB  Ribonuclease, RNase A family, 4  Glucuronic acid epimerase  Protein kinase, cAMP-dependent, regulatory, type II, beta  Selenium binding protein 1  Coiled-coil domain containing 170  Coagulation factor VII (serum prothrombin conversion accelerator)  Androgen receptor  Receptor accessory protein 1  Solute carrier family 44, member 4  Calmegin  Progesterone receptor  Plasminogen activator, tissue  V-myb myeloblastosis viral oncogene homolog (avian)  Carbonic anhydrase XII  Estrogen receptor 1  Cytochrome P450, family 2, subfamily B, polypeptide 7 pseudogene 1  TBC1 domain family, member 9 (with GRAM domain)  Extracellular matrix protein 1  Potassium voltage-gated channel, Isk-related family, member 4  DnaJ (Hsp40) homolog, subfamily C, member 12  Signal peptide, CUB domain, EGF-like 2  Enah/Vasp-like | 0.67  0.67  0.67  0.66  0.66  0.65  0.65  0.64  0.64  0.64  0.62  0.61  0.60  0.59  0.59  0.56  0.55  0.54  0.53  0.53  0.52  0.52  0.52  0.51  0.50 | 1.2×10^-4^  1.5×10^-3^  1.4×10^-2^  9.5×10^-4^  2.0×10^-4^  2.0×10^-3^  2.3×10^-2^  1.3×10^-4^  2.1×10^-3^  9.7×10^-4^  9.6×10^-4^  8.1×10^-3^  2.5×10^-3^  1.6×10^-2^  2.7×10^-2^  1.4×10^-2^  1.2×10^-3^  8.3×10^-8^  7.3×10^-3^  4.6×10^-4^  1.8×10^-3^  7.5×10^-3^  1.9×10^-3^  2.2×10^-2^  1.0×10^-3^ |

(To continue)

| NO. | Gene_symbol | Gene_name | Fold change | P value |
| --- | --- | --- | --- | --- |
| 548  549  550  551  552 | GFRA1  PSD3  C6orf211  GRIA2  NAT1 | GDNF family receptor alpha 1  Pleckstrin and Sec7 domain containing 3  Chromosome 6 open reading frame 211  Glutamate receptor, ionotropic, AMPA 2  N-acetyltransferase 1 (arylamine N-acetyltransferase) | 0.49  0.49  0.49  0.45  0.37 | 3.4×10^-3^  6.1×10^-4^  9.9×10^-3^  1.5×10^-2^  2.3×10^-3^ |
