# Supplementary figures and images for: The expression of CXCL13 and its relation to unfavorable clinical characteristics in young breast cancer
Source: J Transl Med. 2015 May 20;13:168. doi: 10.1186/s12967-015-0521-1 (PMC4471911; doi:10.1186/s12967-015-0521-1)

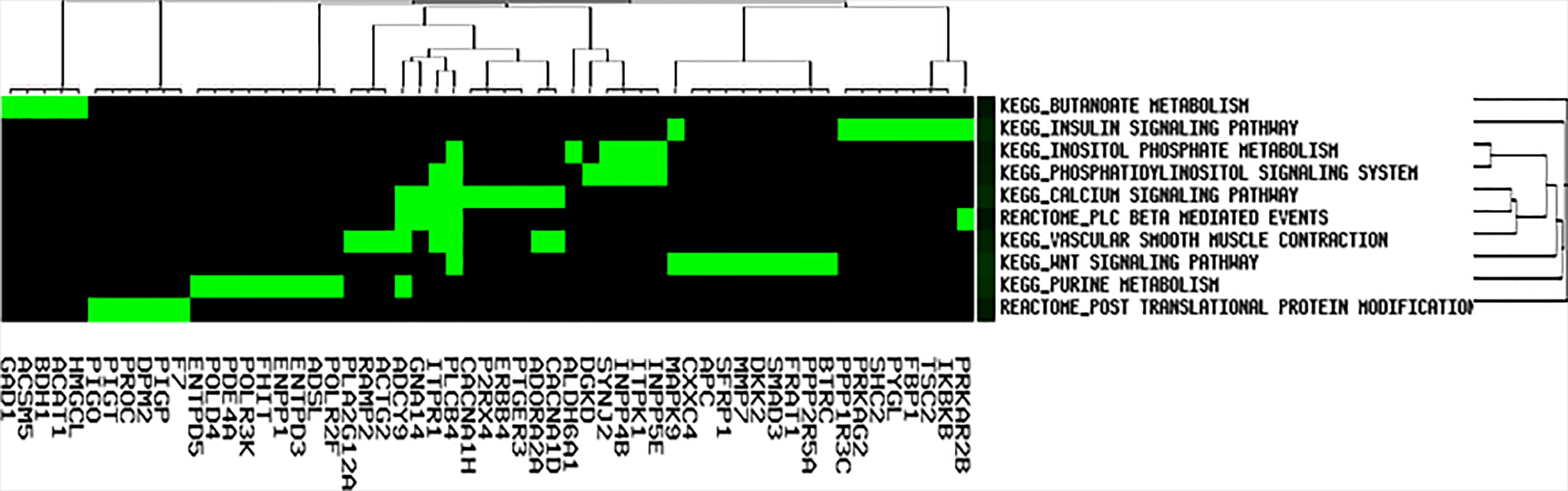

Supplement: Additional file 3: Figure S1. — KEGG pathway analysis of differentially expressed genes. [file 12967_2015_521_MOESM3_ESM.tiff]

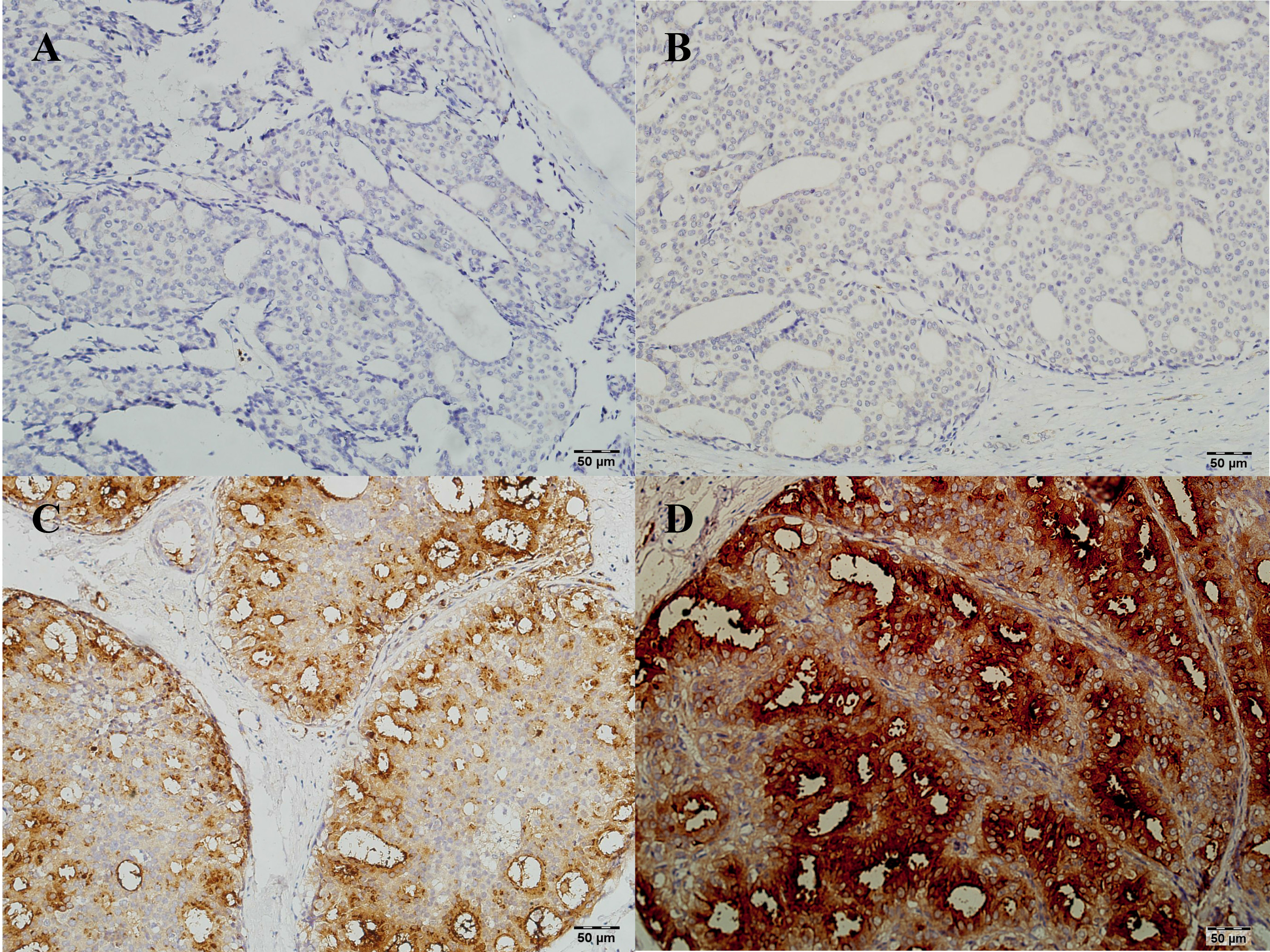

Supplement: Additional file 4: Figure S2. — Immunohistochemistry detection of CXCL13 protein in patients with breast cancer. [file 12967_2015_521_MOESM4_ESM.jpeg]

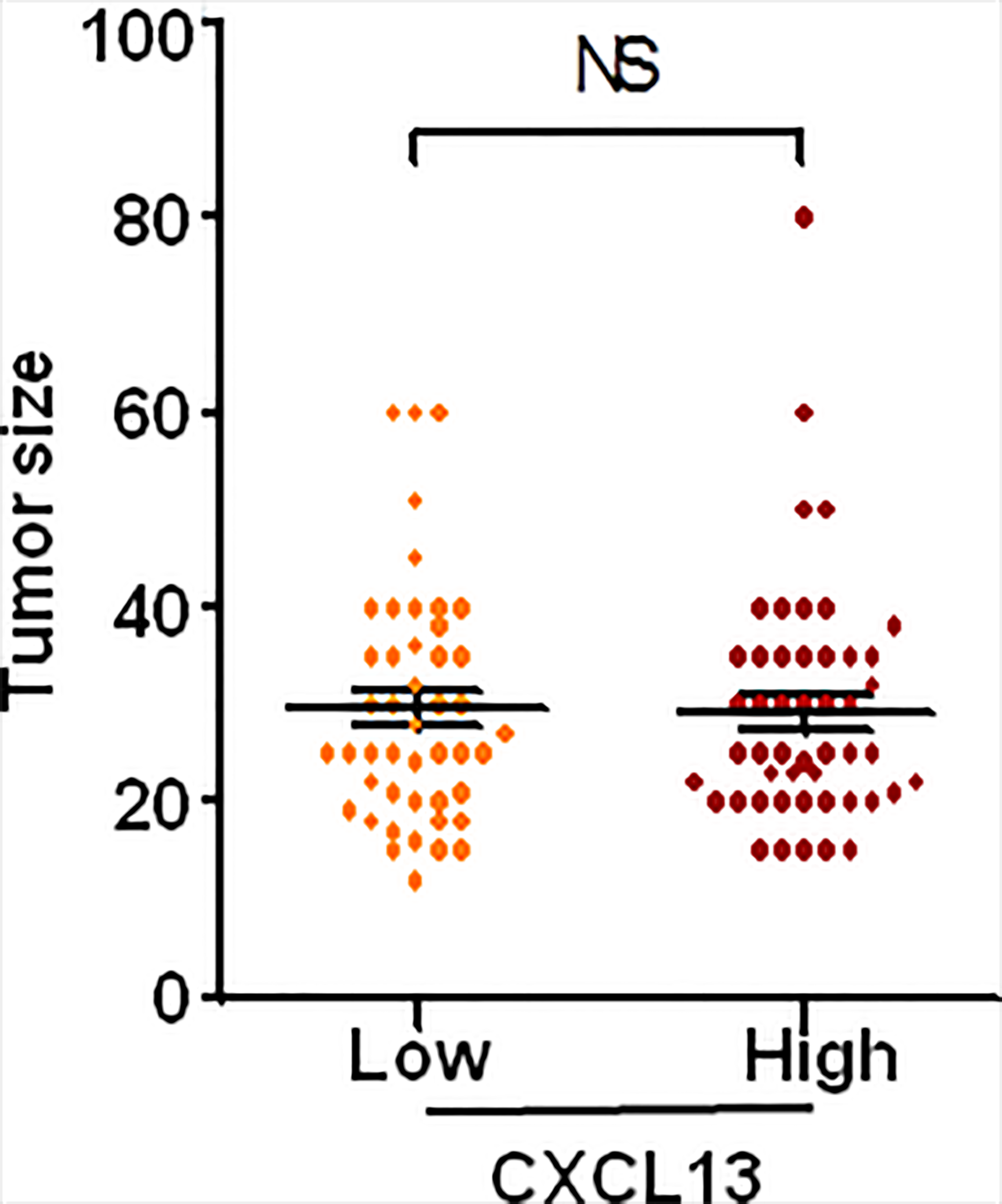

Supplement: Additional file 7: Figure S4. — Analysis of the correlation of CXCL13 expression with tumor size. [file 12967_2015_521_MOESM7_ESM.tiff]
