# Supplementary material for: The expression of CXCL13 and its relation to unfavorable clinical characteristics in young breast cancer
Source: J Transl Med. 2015 May 20;13:168. doi: 10.1186/s12967-015-0521-1 (PMC4471911; doi:10.1186/s12967-015-0521-1)
Supplement: Additional file 6: Table S3. — Linear regression analysis of the correlation of CXCL13 expression with clinicopathological features. [file 12967_2015_521_MOESM6_ESM.doc]

**Supplementary Table S3 Linear Regression Analysis of the Correlation of CXCL13 Expression with Clinicopathological Features**

| Model | Unstandardized Coefficients | | Standardized Coefficients | | | t | Sig. | 95% Confidence Interval for B | | |
| --- | --- | --- | --- | --- | --- | --- | --- | --- | --- | --- |
|  | B | Std. Error |  |  |  |  |  | Lower Bound | | Upper Bound |
| (Constant)  Age  Grade  LN  ER  PR  HER2  Tumor size | 5.874  -6.019  2.503  5.007  -13.689  11.632  -0.177  -0.682 | 8.535  3.015  2.334  2.067  3.494  3.630  1.264  0.530 | | -0.173  0.107  0.219  -0.574  0.481  -0.013  -0.118 | 0.688  -1.997  1.073  2.422  -3.918  3.204  -0.140  -1.288 | | 0.493  0.048  0.286  0.017  0.000  0.002  0.889  0.200 | -11.037  -11.993  -2.121  0.911  -20.612  4.440  -2.681  -1.732 | 22.785  -0.046  7.128  9.103  -6.766  18.824  2.328  0.367 | |

Dependent Variable: CXCL13
